# Pervasive selective sweeps across human gut microbiomes

**DOI:** 10.1101/2023.12.22.573162

**Published:** 2023-12-23

**Authors:** Richard Wolff, Nandita R. Garud

**Affiliations:** 1 Department of Ecology and Evolutionary Biology, UCLA; 2 Department of Human Genetics, UCLA

## Abstract

The human gut microbiome is composed of a highly diverse consortia of species which are continually evolving within and across hosts. The ability to identify adaptations common to many host gut microbiomes would not only reveal shared selection pressures across hosts, but also key drivers of functional differentiation of the microbiome that may affect community structure and host traits. However, to date there has not been a systematic scan for adaptations that have spread across host microbiomes. Here, we develop a novel selection scan statistic, named the integrated linkage disequilibrium score (iLDS), that can detect the spread of adaptive haplotypes across host microbiomes via migration and horizontal gene transfer. Specifically, iLDS leverages signals of hitchhiking of deleterious variants with the beneficial variant, a common feature of adaptive evolution. We find that iLDS is capable of detecting simulated and known cases of selection, and moreover is robust to potential confounders that can also elevate LD. Application of the statistic to ~20 common commensal gut species from a large cohort of healthy, Western adults reveals pervasive spread of selected alleles across human microbiomes mediated by horizontal gene transfer. Among the candidate selective sweeps recovered by iLDS is an enrichment for genes involved in the metabolism of maltodextrin, a synthetic starch that has recently become a widespread component of Western diets. In summary, we demonstrate that selective sweeps across host microbiomes are a common feature of the evolution of the human gut microbiome.

## Introduction

The diverse species that make up the human gut microbiome continually evolve throughout a host’s lifetime. Recent work has shown that rapid adaptation is a hallmark of evolution in the microbiome, as novel mutations often arise and sweep to high frequency within healthy hosts on timescales of days to months [1, 2, 3, 4, 5, 6, 7]. These evolutionary dynamics can have functional consequences for the host, as microbial genetic variants are associated with metabolic capacity, disease susceptibility, and many other host phenotypes [8, 9, 10, 11].

A novel adaptation which appears initially in one host may spread across hosts through strain or phage transmission and subsequent horizontal gene transfer (HGT). The human gut is known to be a hotspot for HGT, allowing alleles which provide a fitness benefit within a host or facilitate the colonization of other hosts to be easily recombined onto new genetic backgrounds [12, 13, 14]. But beyond a handful of particular examples—such as the proliferation of antibiotic resistance genes [15]—the extent to which HGT facilitates the spread of adaptive alleles across hosts is at present unclear, as the ability to systematically detect such adaptations is still emergent.

Should an adaptive allele spread horizontally between hosts in a “gene-specific” selective sweep, the same genomic sequence, or haplotype, will appear in many otherwise distantly related strains collected from many different hosts [12, 16, 17]. Such shared haplotypes will result in distinct signatures of locally elevated linkage disequilibrium (LD), or, correlations among variants that have “hitchhiked” to high frequency with the adaptive allele. However, while elevations in LD have long been leveraged as a signature of selection [18, 19, 20, 21, 22, 23], other evolutionary forces, including demographic contractions and reduced recombination rates, also result in elevations in LD, confounding its use in the discovery of adaptation [24, 25, 26, 27].

**Figure 1:**
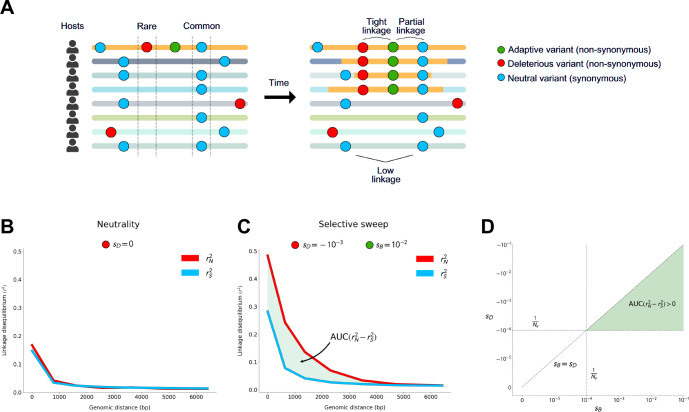
Linkage disequilibrium among common non-synonymous versus synonymous variants during a selective sweep. **(A)** Adaptive variant sweeping across host microbiomes. Each horizontal line represents a bacterial haplotype from a different host’s microbiome. The yellow region of each haplotype represents a fragment that bears an adaptive allele that has recombined onto different lineages’ backgrounds. **(B)** $r_{N}^{2}$ and $r_{S}^{2}$ among common variants under neutrality. **(C)** $AUC\left(r_{N}^{2}-r_{S}^{2}\right)$ among common variants where $s_{D}=-10^{-3}$ and $s_{B}=-10^{-2}$. **(D)** $AUC\left(r_{N}^{2}-r_{S}^{2}\right)$ is expected to be greater than zero when $s_{B}>s_{D}$ and both $s_{D}$ and $s_{B}$ are stronger than the effects of drift ($\frac{1}{N_{e}}$, dashed lines). In this schematic and in all simulations (prior to a demographic contraction), $N_{e}=10^{4}$. See Figures S1 – S3 for common variant $r_{N}^{2}$ and $r_{S}^{2}$ across a comprehensive set of simulated evolutionary scenarios.

One way to control for these non-selective forces is to compare LD among synonymous and non-synonymous variants. While both are subject to the same non-selective forces, synonymous variants are far more likely to be neutral. The vast majority of non-synonymous mutations, by contrast, are deleterious in any population [28], and are always found to be preferentially rare [29, 30]. Hitchhikers that are rare prior to the sweep will exhibit tight linkage with the adaptive mutation during the sweep as they will typically be found only on haplotypes bearing the adaptive mutation. Therefore, we expect non-synonymous variants to be more tightly linked during selective sweeps than synonymous variants in the vicinity of the adaptive locus (Figure 1A).

In this work, we first confirm our hypothesis that deleterious hitchhiking drives an increase of LD among non-synonymous relative to synonymous variants with simulations. We further find that this signal does not manifest under neutrality, as a result of purifying selection alone, or due to low recombination rates or demographic contractions. Next, in a panel of more than 20 prevalent and abundant gut microbiome species, we find that elevations of LD among non-synonymous variants are common at the whole genome level, suggesting that positive selection is widespread. Lastly, we develop a novel statistic leveraging these insights (iLDS, the integrated Linkage Disequilibrium Score) to detect specific loci under selection in these gut microbial species. Application of iLDS to human metagenomic data reveals an abundance of adaptive alleles that have spread across hosts.

## Results

### Positive selection generates elevated linkage disequilibrium among common non-synonymous variants compared to synonymous variants

We first test whether positive selection can drive an excess of LD among non-synonymous variants $\left(r_{N}^{2}\right)$ compared to synonymous variants $\left(r_{S}^{2}\right)$ when deleterious variants hitchhike with a positively selected variant. To do so, we performed forward population genetic simulations of selective sweeps in SLiM 4.0 [31] (Supplementary Section 2). While the beneficial variant and any hitchhikers may be expected to become common in the population, deleterious variants not linked to the adaptive variant should remain rare. Assuming all non-synonymous sites are either subject to purifying selection or are adaptive, we expect non-synonymous variants that become common to either be adaptive or to have hitchhiked with and therefore be tightly linked to an adaptive variant. As a result, we expect that $r_{N}^{2}$ will be elevated relative to $r_{S}^{2}$ specifically among common variants (Figure 1A).

To examine the potential effects of purifying and positive selection on patterns of LD, we analyzed LD among variants that are either rare (minor allele frequency MAF ≤ 0.05) or common (MAF ≥ 0.2) in the broader population, respectively. To quantify whether $r_{N}^{2}$ is significantly elevated over $r_{S}^{2}$, we computed the difference in area under their respective distance decay curves (AUC) (Figure 1C). This test statistic, which we refer to as $AUC\left(r_{N}^{2}-r_{S}^{2}\right)$, allows us to assess differences in total levels of $r_{N}^{2}$ and $r_{S}^{2}$ in a manner that controls for genomic distance (and therefore effective recombination rates) between pairs of alleles (Supplementary Section 1.3).

Before assessing if selective sweeps generate excess LD among common non-synonymous versus synonymous variants, we first determined if this pattern can arise under scenarios of neutrality, purifying selection, or demographic contractions. As expected, under neutrality, we observed that $AUC\left(r_{N}^{2}-r_{S}^{2}\right)$ was not significantly different from zero for either common or rare variants (Figure 1B and S1–S6). Similarly, we found that in populations evolving under purifying selection, in which new non-synonymous mutations experienced purifying selection of strength $\left(s_{D}\right)$ varying from −10^−5^ to −10^−1^ (encompassing a value weaker than the effect of drift $\left(N_{e}s_{D}\ll 1\right.$) to very strong selection $\left(N_{e}s_{D}\gg 1\right)$, common variants failed to produce $AUC\left(r_{N}^{2}-r_{S}^{2}\right)>0$. However, in these scenarios of purifying selection rare variants showed a depression in $r_{N}^{2}$ versus $r_{S}^{2}$ (Figure S5), consistent with both Hill-Robertson interference [32] or epistasis between deleterious variants, as previously observed by [33, 34, 35, 36]. Finally, given that demographic contractions are known to affect patterns of diversity and linkage in ways that closely resemble sweeps [24, 25, 26], we tested if a population bottleneck could lead to a stochastic increase in the frequency of haplotypes bearing particular combinations of linked deleterious variants, and therefore potentially to an elevation of $r_{N}^{2}$ versus $r_{S}^{2}$ among common variants. However, in two demographic scenarios tested, $AUC\left(r_{N}^{2}-r_{S}^{2}\right)$ was not significantly different from zero (Figures S1 – S3).

Next, we tested whether selective sweeps could induce $AUC\left(r_{N}^{2}-r_{S}^{2}\right)>0$ among common variants. To do so, we introduced a novel, beneficial mutation to a population already evolving under purifying selection, and allowed it to rise to intermediate (50%) frequency. The strength of beneficial selection $\left(s_{B}\right)$ ranged from nearly-neutral (10^−5^) to strongly beneficial (10^−1^). First, regardless of $s_{D},$ $r_{N}^{2}$ and $r_{S}^{2}$ among common variants generally increased monotonically with $s_{B}$, reflecting the decrease in the expected time for the sweeping variant to reach intermediate frequency. Second, we found that selective sweeps can in fact produce $AUC\left(r_{N}^{2}-r_{S}^{2}\right)>0$; however, this pattern only manifests under particular combinations of $s_{B}$ and $s_{D}$. Specifically, the strength of purifying selection must exceed drift (i.e. $s_{D}>\frac{1}{N_{e}}$), and the strength of positive selection must exceed that of purifying selection $\left(s_{B}>s_{D}\right)$ (Figures 1D and S2). Additionally, $AUC\left(r_{N}^{2}-r_{S}^{2}\right)$ increased with the strength of $s_{B}$ and $s_{D}$, as well as with the rate of recombination (Figures S1 – S3). Moreover, $r_{S}^{2}$ remained elevated over $r_{N}^{2}$ among rare variants during the selective sweep, provided purifying selection exceeded drift (Figure S4 – S6). Thus, when a population experiences both purifying and positive selection, we expect to see differences between synonymous and non-synonymous LD among both rare and common variants.

### Elevation of LD among non-synonymous variants in gut commensal species

Having established in simulations that LD between non-synonsymous variants can be elevated relative to synonymous variants primarily due to selective sweeps, we next quantified $r_{N}^{2}$ and $r_{S}^{2}$ across host metagenomes to assess if this signature of positive selection is observed at a genome-wide scale in gut microbiome species. To do so, we analyzed data from metagenomic samples of 693 individuals from North America, Europe, and China [37, 38, 39, 40]. To identify single nucleotide polymorphisms (SNPs) from these samples, we aligned shotgun reads to a database of reference genomes using MIDAS [41] (Supplementary Section 3). We showed previously that samples in which a single dominant strain of a species is present can be confidently ‘quasi-phased’ such that pairs of alleles can be assigned to the same haplotype with low probability of error, and that subsequently LD can be computed between these pairs of alleles [1]. With this quasi-phasing approach, we extracted 2641 haplotypes belonging to 30 species across the 693 individuals we examined. Some of the species examined exhibit considerable population structure, with strong gene flow boundaries between clades, so we focused our analyses only on haplotypes belonging to the largest clade of each species (Supplementary Section 3.6) [1, 12].

**Figure 2:**
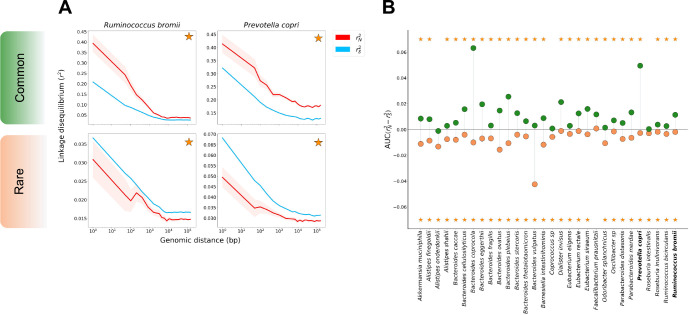
$r_{N}^{2}\;and\;r_{S}^{2}$ measured in prevalent commensal gut microbiota. **(A)** Decay in LD among common (MAF ≥ 0.2) (top) and rare (MAF ≤ 0.05) (bottom) variants for the species *Ruminococcus bromii* and *Prevotella copri*. Both species show significant differences between $r_{N}^{2}$ and $r_{S}^{2}$ for common and rare variants, as denoted by the orange star. **(B)** $AUC\left(r_{N}^{2}-r_{S}^{2}\right)$ among rare (orange) and common (green) alleles for 30 gut commensal bacteria species. Among rare variants, $AUC\left(r_{N}^{2}-r_{S}^{2}\right)$ is significantly negative for all but one species (yellow stars, at bottom). Among common variants, $AUC\left(r_{N}^{2}-r_{S}^{2}\right)$ is significantly positive in 27/30 of species (yellow stars, at top).

Shown in Figure 2A are examples of genome-wide $r_{N}^{2}$ and $r_{S}^{2}$ for the species *Ruminococcus bromii* and *Prevotella copri*. Among both rare and common variants, $r_{S}^{2}$ and $r_{N}^{2}$ decay with increasing distance between pairs of genomic loci, as expected for recombining species. The rate of decay differs among species; however, for all species, LD appears to eventually saturate to some roughly constant value. In *R. bromii*, for instance, both rare and common variant LD appear to saturate around ~10Kb. In Supplementary Section 4.1, we show how the initial decay and eventual saturation of LD can be related to an underlying model of recombination, which in turn can be used to infer the mean tract length of horizontally transferred segments for each species.

For both species in Figure 2A, $AUC\left(r_{N}^{2}-r_{S}^{2}\right)$ is significantly greater than zero among common variants and less than zero among rare variants. More broadly, across the 30 species analyzed in this study, $AUC\left(r_{N}^{2}-r_{S}^{2}\right)$ is significantly greater than zero among common variants in 27/30 species. Among rare variants, $AUC\left(r_{N}^{2}-r_{S}^{2}\right)$ was significantly less than zero for all but one species (Figure 2B). Together, these patterns of LD among synonymous and non-synonymous variants are consistent with widespread purifying and positive selection acting on non-synonymous sites in these species.

Finally, we more finely examined the dependence of $AUC\left(r_{N}^{2}-r_{S}^{2}\right)$ on allele frequency. As purifying selection drives deleterious variants to low frequencies and positive selection tends to elevate allele frequencies, we expect to observe a generally positive relationship between allele frequency and $AUC\left(r_{N}^{2}-r_{S}^{2}\right)$ if both purifying and positive selection affect these populations. In Figure S13, we see that $AUC\left(r_{N}^{2}-r_{S}^{2}\right)$ universally increases with allele frequency, as expected. Additionally, we see that $AUC\left(r_{N}^{2}-r_{S}^{2}\right)$ flips from negative to positive at a frequency of approximately 0.05 in most species. It is possible that the majority of non-synonymous variants with allele frequencies below this threshold are deleterious, while those with allele frequencies above this threshold are more likely to be either beneficial themselves or tightly linked to a beneficial variant.

### Detecting recombinant selective sweeps with iLDS

Genome-wide patterns of LD among synonymous and non-synonymous variants indicate that selection—both positive and purifying—is pervasive at the nucleotide level in gut microbiome species. While only a minority of intermediate frequency non-synonymous sites are likely adaptive, positive selection at these sites is evidently strong enough to create highly significant genome-wide linkage patterns. To identify these specific adaptive loci, we developed a novel statistic—the integrated Linkage Disequilibrium Score (iLDS)—which detects genomic regions exhibiting both $AUC\left(r_{N}^{2}-r_{S}^{2}\right)>0$ and elevated LD relative to the genomic background. By combining these sources of information, we identify regions which have elevated LD due to positive selection and not other non-selective forces.

To detect specific genomic regions under selection, iLDS is calculated in sliding windows across a genome. To calculate iLDS in a genomic window, we first determine $AUC\left(r_{N}^{2}-r_{S}^{2}\right)$ among common SNVs (MAF ≥ 0.2) within the window. To augment our ability to detect selection, we also identify windows with outlier LD values, which is expected to be elevated in selective sweeps. To do so, we measure local elevation in overall LD in the window compared to the genomic background by computing the area under the LD curve between all intermediate frequency variants in the same window (i.e. $AUC\left(r^{2}\right)$), irrespective of whether they are synonymous or non-synonymous, and then normalize this quantity by the average genome-wide LD over the distance defined by the window: $AUC(r_{\mathrm{genome}-\mathrm{wide}}^{2})$. The statistic is then defined as:

(1) $$iLDS=AUC(r_{N}^{2}-r_{S}^{2})\times (AUC(r^{2})/AUC(r_{\mathrm{genome}-\mathrm{wide}}^{2}))$$

In essence, $AUC(r^{2})/AUC(r_{\mathrm{genome}-\mathrm{wide}}^{2})$ quantifies the increase in total LD within the window relative to the expected level of LD across the whole genomic background for a region of the same size, while $AUC\left(r_{N}^{2}-r_{S}^{2}\right)$ quantifies the local elevation in linkage among non-synonymous variants relative to synonymous variants. Both of these terms are expected to be elevated during a sweep; however, iLDS should not be elevated in regions where total linkage is high due to non-selective factors, as $AUC\left(r_{N}^{2}-r_{S}^{2}\right)$ will remain near zero in such regions. LLDS is determined to be significant if both $AUC\left(r_{N}^{2}\right)$ is significantly greater than $AUC(r_{S}^{2})$, and $AUC(r^{2})$ is significantly greater than $AUC(r_{\mathrm{genome}-\mathrm{wide}}^{2})$ (Supplementary Section 4.2).

We first tested iLDS’s capacity to correctly detect simulated selective sweeps, as well as its potential for misclassifying genomic regions with elevated LD arising from demographic contractions as selective sweeps. To do so, we evaluated the true positive rate (TPR) and false positive rate (FPR) of the statistic in the simulations described above (Supplementary Section 4.3). Among positive selection scenarios, iLDS was frequently significant when $s_{B}>s_{D}$ and $s_{D}>\frac{1}{N_{e}}$ and had increasing accuracy for stronger sweeps. In particular, iLDS had a true positive rate of 85% when $s_{B}=10^{-2}$ and $s_{D}=10^{-3}$, and 100% when $s_{B}=10^{-1}$ and $s_{D}=-10^{-3}$ or −10^−2^ (Figure S7). In the populations which had undergone demographic contractions, by contrast, iLDS was almost never significant regardless of the strength of purifying selection—the FPR was 0% for the vast majority of parameter combinations and only when recombination was very weak and purifying selection was weaker than drift did the FPR reach a maximum of ~1% (Figure S8). These simulation results indicate that overall, iLDS is capable of correctly identifying sweeps when $s_{B}$ is sufficiently strong and exceeds the strength of purifying selection and very rarely identifies non-sweeps as sweeps.

### iLDS reveals pervasive selective sweeps in gut bacteria

We next applied iLDS to gut bacteria. To do so, it is first necessary to define genomic windows to calculate iLDS in. The window size should ideally be large enough that genome-wide LD can be expected to fully decay by the edges of the window, but not so large that the footprint of the sweep is very small relative to the size of the window. To determine this species-specific window size in the bacteria examined here, we estimated a typical upper bound on the size of a horizontally transferred tract $\left(l_{DD}\right)$ under an idealized model of HGT (Supplementary Section 4.1). LD should fully decay at approximately $l_{DD}$ as linkage between fragments separated by greater than this distance is always broken by recombination, while variants which are closer may be transferred together horizontally, preserving linkage. By visual inspection, we found that the inferred value of $l_{DD}$ did in fact correspond to the point at which LD fully decayed among common synonymous variants in the data (Supplementary Section 4.1, Figure S17, Table S4). To ensure that each window contains both an adequate and comparable number of synonymous and non-synonymous variants with which to calculate $r_{N}^{2}$ and $r_{S}^{2}$ curves, we employed a SNP based windowing approach as opposed to a base-pair defined window. Specifically, we defined each window to consist of the average number (for that species) of consecutive non-synonymous, intermediate frequency SNPs (MAF ≥ 0.2) spanning $l_{DD}$ (Table S3).

**Figure 3:**
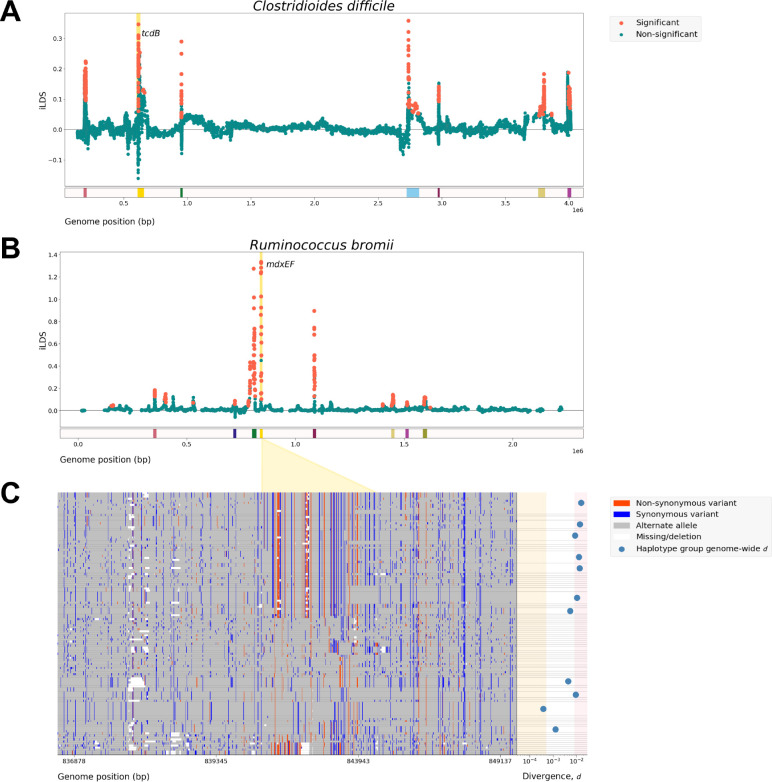
Recombinant selective sweeps in gut bacteria. **(A)** iLDS scan in *Clostridioides difficile*. Each point corresponds to an iLDS value for a given genomic window centered around a single intermediate frequency non-synonymous SNP. Significant windows are colored orange, while nonsignificant windows are colored green. The locations of peaks are shown as colored bars below the scan. **(B)** iLDS scan in *R. bromii*. **(C)** Haplotype plot of a ~10Kb region surrounding the sweep candidate mdxEF genes. Non-synonymous variants are colored red, while synonymous variants are colored blue, and missing sites are colored white. Horizontal lines separate strains into haplotype groups that are genetically identical at mdxEF. Haplotypes are ordered based on their genetic distance to the largest haplotype at the mdxEF locus. To aid in visualization, intermediate frequency variants (MAF > 0.2) are colored based on the identity of the first strain in the first haplotype group (i.e. the top row), while the color of all other sites is determined by minor allele at that position. The alternate allele at each site assigned by this polarization scheme is colored gray. At right, mean genome-wide divergence d within each haplotype group containing three or more strains is shown with blue dots, while the pink region shows the typical range of d (within a factor of two of the mean), and the orange region denotes the region $d<5\times 10^{-4}/bp$, indicative of close-relatedness among strains.

Next, to assess the ability of iLDS to detect known instances of positive selection in a natural population, we applied iLDS to a set of 257 isolates of *Clostridiodes difficile* (Figure 3A, Supplementary Section 6), an enteric pathogen. *C. difficile* has experienced recombination mediated selective sweeps at the *tcdB* locus, which encodes the its chief virulence factor, toxin B [42, 43]. In the majority of windows, iLDS remains close to 0, as expected in the absence of positive selection. However, the value of the statistic peaks sharply in several regions across the genome, and these peaked regions contain large numbers of significant iLDS values. Since adjacent windows may belong to the same selective event, we clustered groups of significant windows into a peak if the SNPs they were centered around were both physically close and tightly linked to one another, as would be expected following a selective sweep (Supplementary Section 4.4). The second highest peak in the scan coincides with the *tcdB* locus, confirming that our scan can indeed recover known instances of positive selection mediated by HGT. Other peaks may be candidates for selection as well, such as the leftmost peak, which is made up of genes in the flagellar operon *fli* that have also been previously reported to be under positive selection in *C. difficile* [44]. The locations, names, and annotations of all genes found within a peak for *C. difficile* and all other species can be found in Supplementary Table S4.

Finally, we applied the scan to 23 gut microbiome species shown in Figure 2B whose reference genomes were not fragmented into a large number of contigs, which otherwise produced sparse, unreliable scans (Supplementary Section 3.5). Across these 23 species, we recovered 169 peaks, with a median of 5 peaks per species. All species exhibited at least 1 peak (Table S4, Figure S14).

Figure 3B shows the iLDS scan for one of these species: *R. bromii*, a common gut commensal species known to be critical for the digestion of resistant starches in the colon [45]. We detected a total of 8 distinct peaks in this species, in line with the typical number of peaks observed per species in our dataset. The tallest peak coincides with the genes mdxEF and mdxEF, which are ATP-binding cassette transporters involved in metabolizing maltodextrin [46]. *R. bromii* was not alone in exhibiting peaks containing genes related to maltodextrin metabolism: 3 other species (*E. rectale*, *E. siraeum*, and *B. cellulosilyticus*) also had peaks overlapping maltodextrin-related genes (Table S4). A gene enrichment analysis showed that maltodextrin-related genes were the only class of genes enriched for signatures of selection in our dataset, after controlling for false discovery (Supplementary Section 5). Several other classes of genes were found to be under selection in multiple species, including two-component sensor histidine kinases and SusC/SusD polysaccharide utilization loci that have been previously observed to evolve within hosts [2, 7]; however, they were not statistically overrepresented among selected genes relative to their overall prevalence in core genes.

To assess if the peaks in the R*. bromii* scan replicate in an independent dataset, we repeated our analysis in a collection of isolates collected from healthy adults [47]. Both of the highest peaks from HMP (including mdxEF) also appeared as peaks in the isolate dataset, though the remaining 6 smaller peaks did not (Figure S16, Supplementary Text 6).

The putatively adaptive regions in *R. bromii* show evidence of extensive, recent horizontal gene transfer. To illustrate this, Figure 3C shows the genomic diversity of a ~10Kb window surrounding the ~3Kb mdxEF locus (gold region), in which strains are grouped by haplotype identity at mdxEF. At right we show genome-wide nucleotide divergence d at fourfold synonymous sites (that is, sites where no nucleotide change induces a change in amino acid) among each group of strains which are identical across mdxEF. By visual inspection, it is clear that the top half of the figure is dominated by 26 haplotypes (comprising 48/128 strains) which are much more similar one another $\left(d=3.06\times 10^{-3}/bp\right)$ than the remaining 50 haplotypes, whose d of 1.73×10^−2^/bp is quite close to genome-wide average $d=1.69\times 10^{-2}/bp$ (Figure S15). However, haplotype identity among those lineages harboring identical mdxEF haplotypes decays rapidly in the regions immediately flanking mdxEF, with little apparent linkage between variants found on either end of the extended window. For each of the 26 haplotypes highlighted above, genome-wide pairwise divergence d falls within a factor of 2 of the average d for all pairs of strains (blue dots, Figure 3C). This is in contrast to a set of lineages which are closely related genome-wide ($d<5\times 10^{-4}/bp$, see Supplemental Section 3.6) that are not only identical at mdxEF but also maintain substantial haplotype identity in the flanking regions. Together, these patterns are highly consistent with the horizontal transfer of this segment in a recombinant sweep.

## Discussion

In this paper, we perform the first comprehensive scan for sweeps that have spread across human microbiomes. Although previous work has found evidence of genomic fragments shared across hosts in a manner inconsistent with neutrality [12], we definitively establish here that many fragments are spreading via gene-specific selective sweeps. To do so, we develop a novel statistic, iLDS, that is robust to many evolutionary forces which traditionally confound selection scans, including purifying selection, demographic contractions, and reduced recombination rates [24, 25, 26], and show that the spread of adaptive fragments across hosts in gene-specific sweeps is a pervasive feature of the evolution of ~20 of the most prevalent and abundant commensal microbiome species in a cohort of healthy, Western adults.

The iLDS statistic that we develop to discover sweeps across host microbiomes is versatile enough to be applied to any recombining species. Demonstrating this, we found that iLDS is capable of recovering known selective sweeps not only in recombinant bacteria (like the pathogen *C. difficile*), but also in *Drosophila melanogaster* (Figure S21, Supplementary Section 7). iLDS may have power in diverse systems because it exploits a common signature associated with selective sweeps: deleterious variants hitchhiking to high frequency with a beneficial variant. To our knowledge, the tight linkage of beneficial variants with hitchhiking deleterious variants, which has been shown to be a common feature of evolution both in theory and in numerous systems [48, 49, 50, 51, 52], has not been explicitly incorporated into any selection scan statistic. By contrast, current LD-based methodology for detection of sweeps instead relies more generically on signatures of hitchhiking which are not unique to selective sweeps [18, 19, 20, 21, 22, 23]. Building on these previous scans, iLDS quantifies not only the overall increase of LD but also the excess of LD among non-synonymous variants compared to synonymous variants, leveraging additional power that deleterious hitchhikers provide in sensitively detecting sweeps.

Others have also shown that elevated linkage among common non-synonymous variants relative to synonymous variants can be a signature of adaptation; however, the connection with deleterious hitchhiking had not previously been noted. Stolyarova *et al.* [53] found that sign epistasis generated elevated LD among non-synonymous variants in the highly polymorphic fungus *Schizophyllum commune*, while Arnold *et al.* [54] concluded that epistatic interactions were not necessary to generate this signal in *Neisseria gonorrhoeae*, and that adaptive inter-specific HGT of short genomic fragments bearing multiple positively selected non-synonymous alleles was the likely driving factor. We emphasize that our findings are fully consistent with those of Stolyarova *et al.* and Arnold *et al*. But crucially, our results suggest that elevated linkage among common non-synonymous variants is not by itself sufficient to establish that all such variants are adaptive. We find instead that it is highly likely some proportion of common non-synonymous polymorphisms will be deleterious hitchhikers in any adapting population, with this proportion growing, paradoxically, as the strength of positive selection increases. Establishing precisely what level of non-synonymous diversity is truly inconsistent with hitchhiking—a quantity which will at minimum depend on the distribution of deleterious fitness effects, the strength of positive selection, and the ratio of recombination to mutation rate—is an avenue of future research.

iLDS is powerful in detecting strong selective sweeps in the human gut microbiome, therefore is potentially biased towards the most extreme cases of adaptation. Specifically, we found that iLDS had power to detect sweeps when the strength of positive selection exceeds that of purifying selection $\left(s_{B}>s_{D}\right)$, and when the strength of purifying selection exceeds drift $\left(s_{D}>\frac{1}{N_{e}}\right)$. Both very weak sweeps and sweeps in regions of the genome in which purifying selection is relaxed are unlikely to be detected with iLDS. Indeed, absent purifying selection, iLDS will fail to detect even the very strongest sweeps. However, we note that purifying selection is a pervasive feature of bacterial genomes [1, 55, 56, 57, 58], and it seems unlikely that there would be large genomic regions devoid of variants that are under purifying selection.

Additionally, iLDS is best suited to detect recombinant, gene—specific selective sweeps. Genome-wide sweeps—for instance, a rapid clonal expansion of a single lineage in an asexually evolving population—should produce globally elevated LD among common variants linked on the selected haplotype, and consequently little variation in either $AUC\left(r_{N}^{2}-r_{S}^{2}\right)$ or $AUC\left(r^{2}\right)/AUC(r_{\mathrm{genome}-\mathrm{wide}}^{2})$ across windows. At the other extreme, parallel, recurrent adaptations which arise in different hosts and do not spread between hosts will also be undetectable with iLDS, as each adaptive sweeping variant is likely to carry private, minimally overlapping sets of hitchhikers in each distinct host. While both modes may contribute to adaptation in differing degrees in different species, we emphasize that iLDS is designed to detect gene-specific sweeps mediated by recombination. We note, however, that the selective sweeps we found need not have arisen from a single mutational origin: rather, these adaptations may have arisen multiple times independently in different hosts and subsequently spread simultaneously via migration and recombination.

But while iLDS may fail to detect all true positives, it is also robust to known sources of false positives that previous scans for selection may be sensitive to, including demographic contractions. We found that FDR values for iLDS are zero for most evolutionary scenarios, and it is only when the strength of purifying selection is smaller than the effect of drift and when recombination rates are extremely low that FDRs can achieve rates up to ~1%. These conditions are unlikely in the microbiome, as recombination is a ubiquitous and powerful force in the evolution of gut commensals and as stated above, it is unlikely that bacterial genomes experience such weak purifying selection. However, we acknowledge that finer resolution maps of purifying selection and recombination across these genomes will be informative for understanding the extent to which sweeps are mis-inferred due to these processes. Additionally, despite the extremely low rate of false positives observed in simulations, we also employed a conservative approach to identifying “peaks” (i.e. individual selective sweeps) in our scans, requiring that each peak be supported by multiple significant windows centered around variants with tight linkage to one another. iLDS is thus insulated from producing false positive inferences of selection at multiple levels.

Some of the candidate sweeps we found may be related to changing Western lifestyles. In particular, we saw that genes related to the metabolism maltodextrin—a synthetic starch which has increased dramatically in abundance in Western diets in recent years [59, 60]—appeared to be under selection in several species. As consumption of maltodextrin has been implicated experimentally in the onset of colitis [61, 62], these selective sweeps may have implications for human health. More broadly, future work in which systematic scans of adaptation are performed in a variety of cohorts may reveal genetic loci in the microbiome that are especially relevant to certain human conditions. By being able to detect adaptations in gut microbiomes in a high throughput manner, we can look for patterns that are common to one cohort and absent from another. In doing so, we may gain mechanistic insight into how microbiome genotypes confer disease, improving our ability to diagnose and treat such conditions, and potentially allowing us to deploy existing natural, adaptive variation in the design of rational probiotics.

## Supplementary Material

Supplement 1

Supplement 2
