# Supplementary material for: Pervasive selective sweeps across human gut microbiomes
Source: bioRxiv. 2023 Dec 23:2023.12.22.573162. Preprint. [Version 1] doi: 10.1101/2023.12.22.573162 (PMC10769429; doi:10.1101/2023.12.22.573162)
Supplement: Supplementary file 1 [file media-1.pdf]

Supplementary materials

Table of Contents

List of Supplementary Tables

Table S1: Metadata for metagenomic samples. This table contains a list of the 1,013 shotgun metagenomic samples collected from 693 unique, healthy individuals which we used in this study, and were originally analyzed by Garud & Good (2019) [1]. This includes 250 individuals from Lloyd-Price (2017) (accession numbers PRJNA48479 and PRJNA275349) [2], 250 from Xie *et al* (2016) (accession number PRJEB9576) [3], 185 from Qin *et al* (2012) (accession number PRJNA422434) [4], and 8 from Korpela *et al* (2018) (accession number PRJEB24041) [5]. For each sample used, the subject identifier, sample identifier, accession number, country of the study, continent of the study, and visit number are included, as well as the study from which the sample was drawn.

Table S2: Clade definitions. This table contains the top-level clades which were manually defined for this dataset by Garud & Good (2019) [1]. Each row details the species and clade label of a single QP sample.

Table S3: iLDS scan parameters. This table shows the parameters used to perform the iLDS scans. In particular, for each species, it shows the tract length l_r, decay distance l_{DD}, window size, number of bins per window, and the total number of intermediate frequency variants non-synonymous variants present genome-wide. For further methodological details on how these quantities were obtained, see Supplementary Section 4.1.

Table S4: Peaks. This table gives the locations and annotations of all intermediate frequency non-synonymous variants inferred to be in a peak of iLDS. An intermediate frequency non-synonymous variant is considered to be in a peak if it is the center of a significant window and is both tightly linked and physically proximate to variants lying at the center of other significant windows (for further details on our approach to obtaining peaks, which are each meant to reflect a single selective sweep, see Supplementary Section 4.4). The columns of the table are:

- **Species**: Name of the species
- **Peak number** the unique peak to which the variant belongs (peaks are numbered from left to right for each species)

- **Contig**: the contig the variant lies on (in the case of peaks for *Drosophila melanogaster*, this is the chromosome)
- **Gene**: the gene the variant lies in
- **SNV position (contig)**: the position of the variant along the given contig
- **SNV position (genome)**: the inferred position of the variant along the entire genome (used only for plotting purposes)
- **Gene product**: the gene protein product
- **GO annotation**: the gene ontology annotation
- **Pathway**: the inferred pathway type the gene lies in
- **Data type**: the dataset the scan was performed on (QP metagenomes, UHGG, or Drosophila)
- **iLDS**: value of iLDS for the variant

Table S5: Metadata for isolate samples. Metadata for the isolates of *R. bromii* and *C. difficile* used in this study. These sequences, and the metadata presented in this table, were downloaded from UHGG [6]. The SNV catalogs used here are available from the MGnify FTP site (http://ftp.ebi.ac.uk/pub/databases/metagenomics/mgnify_genomes/).

List of Figures

S1 r_N^2 and r_S^2 measured among common variants in a range of simulated evolutionary scenarios, where $\rho = 10^{-5}$ 6
S2 r_N^2 and r_S^2 measured among common variants in a range of simulated evolutionary scenarios, where $\rho = 10^{-6}$ 7
S3 r_N^2 and r_S^2 measured among common variants in a range of simulated evolutionary scenarios, where $\rho = 10^{-7}$ 8
S4 r_N^2 and r_S^2 measured among rare variants in a range of simulated evolutionary scenarios, where $\rho = 10^{-5}$ 9

S5 r_N^2 and r_S^2 measured among rare variants in a range of simulated evolutionary scenarios, where $\rho = 10^{-6}$ 10
S6 r_N^2 and r_S^2 measured among rare variants in a range of simulated evolutionary scenarios, where $\rho = 10^{-7}$ 11
S7 True positive rate of iLDS in simulated selective sweeps. 12
S8 False positive rate of iLDS in simulated demographic contractions 13
S9 Number of QP samples by species 14
S10 r_N^2 and r_S^2 measured in prevalent commensal gut microbiota for common variants . 15
S11 $(r_N^2 - r_S^2)$ measured in prevalent commensal gut microbiota for common variants. . 16
S12 r_N^2 and r_S^2 measured in prevalent commensal gut microbiota for rare variants 17
S13 AUC($r_N^2 - r_S^2$) measured in prevalent commensal gut microbiota for varying allele frequencies 18
S14 iLDS scans for all QP species analyzed not shown in Main Text 19
S15 Genomic diveristy at the *mdxEF* locus versus genome-wide for the species *R. bromii* 20
S16 iLDS scan for *R. bromii*, UHGG 21
S17 Estimates of l_{DD} for common commensal gut microbiota 22
S18 Schematic of method for inferring l_r and l_{DD} 23
S19 Comparison of l_r and l_{DD} estimates with those of Liu & Good (2023) 24
S20 Decay of LD among common variants in *D. melanogaster*. 25
S21 iLDS scans in *D. melanogaster* 26

Supplementary Text

1 LD **27**
1.1 LD calculations 27
1.2 AUC 27
1.3 LD confidence intervals 27

2 Simulations **29**
2.1 Burn-in/mutation-selection balance 30
2.2 Demographic contractions 30
2.3 Partial sweep 31

3 Metagenomic pipeline **32**
3.1 Overview 32
3.2 Data 32
3.3 Estimation of species, gene, and SNV content of shotgun metagenomic samples . . 32
3.3.1 Estimation of species content 32
3.3.2 Estimation of CNV content 33
3.3.3 Estimation of SNV content 33
3.4 Quasi-phasing 34
3.5 Inclusion criteria 34
3.6 Population structure 34

4 iLDS **36**
4.1 Determining the window size for computing iLDS 36
4.1.1 Comparison of tract length estimates with previous work 38
4.2 Identification of analysis windows with significant iLDS values 39
4.3 Testing iLDS on simulations 39
4.4 Peak calling 41

5 Gene enrichment analysis **43**

6 Analyses with isolates **44**
6.1 Overlap of peaks in the *R. bromii* scans performed in metagenomic QP data and UHGG data 44

7 Analyses with *Drosophila melanogaster* **46**

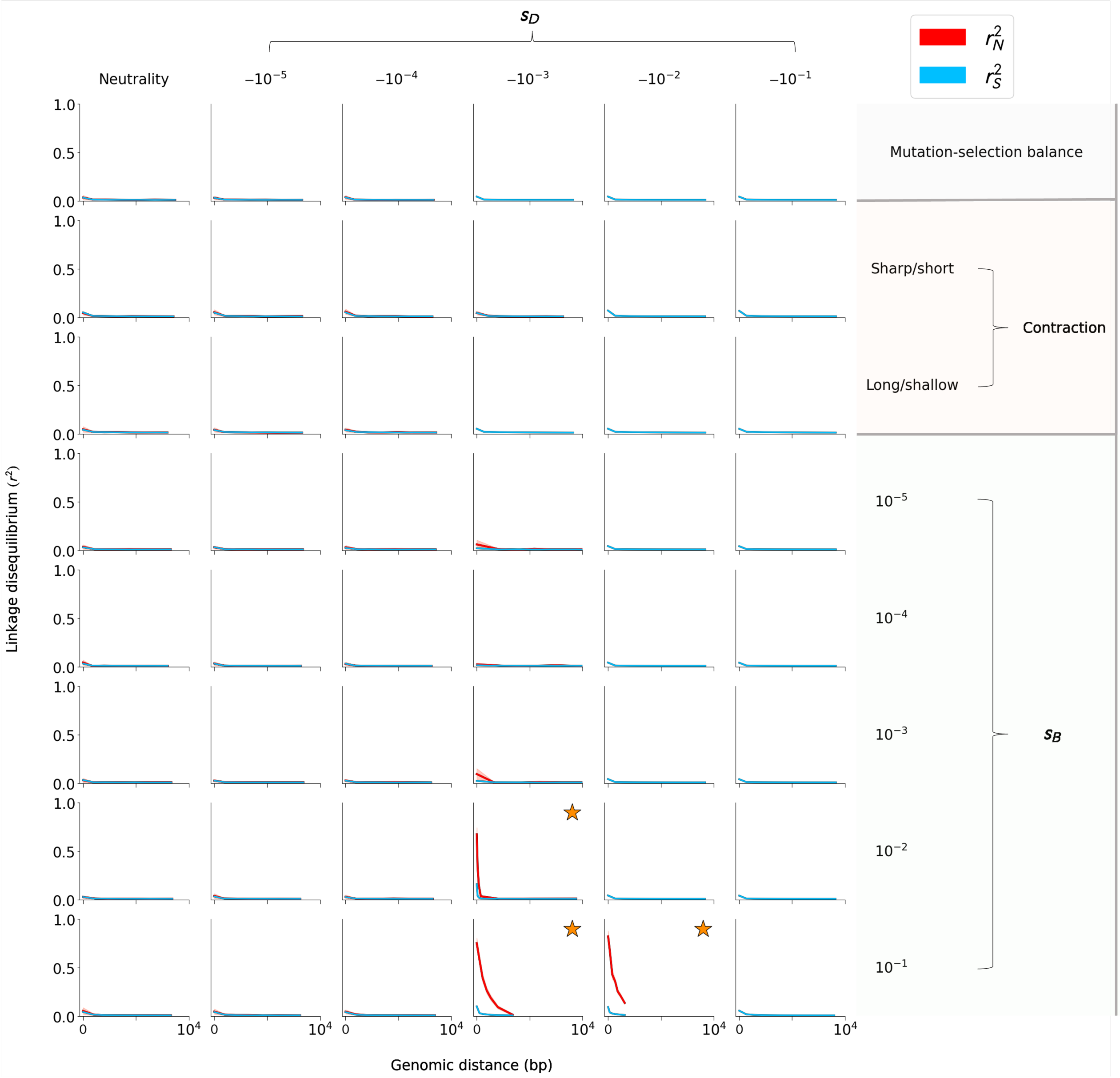

Figure S1: r_N^2 and r_S^2 measured among common variants in a range of simulated evolutionary scenarios, where $\rho = 10^{-5}$. Populations of size $N_e = 10^4$ were simulated with a range of deleterious selection coefficients (s_D), and with $\rho/\mu = 0.1$ (see Figures **S2** and **S3** for $\rho/\mu = 1$ and $\rho/\mu = 10$) Top row: r_N^2 and r_S^2 measured in populations at mutation-selection balance. When $s_D \geq 10^{-2}$, no intermediate frequency non-synonymous variants were observed, and therefore no r_N^2 curves are plotted in these columns. Second and third rows: r_N^2 and r_S^2 measured in populations experiencing a sharp, short population size contraction (second row) and a long, shallow contraction (third row). Fourth row on: r_N^2 and r_S^2 measured in populations experiencing a selective sweep. A total of 250 replicate burn-in populations were simulated until mutation-selection balance had been reached ($10N_e$ generations), followed by five replicates for each burn-in population for the bottleneck and selective sweep scenarios (Section 2). Orange stars denote significantly elevated r_N^2 compared to r_S^2. Under the strongest selective conditions ($s_B = 10^{-1}, s_D = \{-10^{-3}, -10^{-2}\}$), nearly all common deleterious variants were physically close to the adaptive variant, resulting in apparently truncated LD curves.

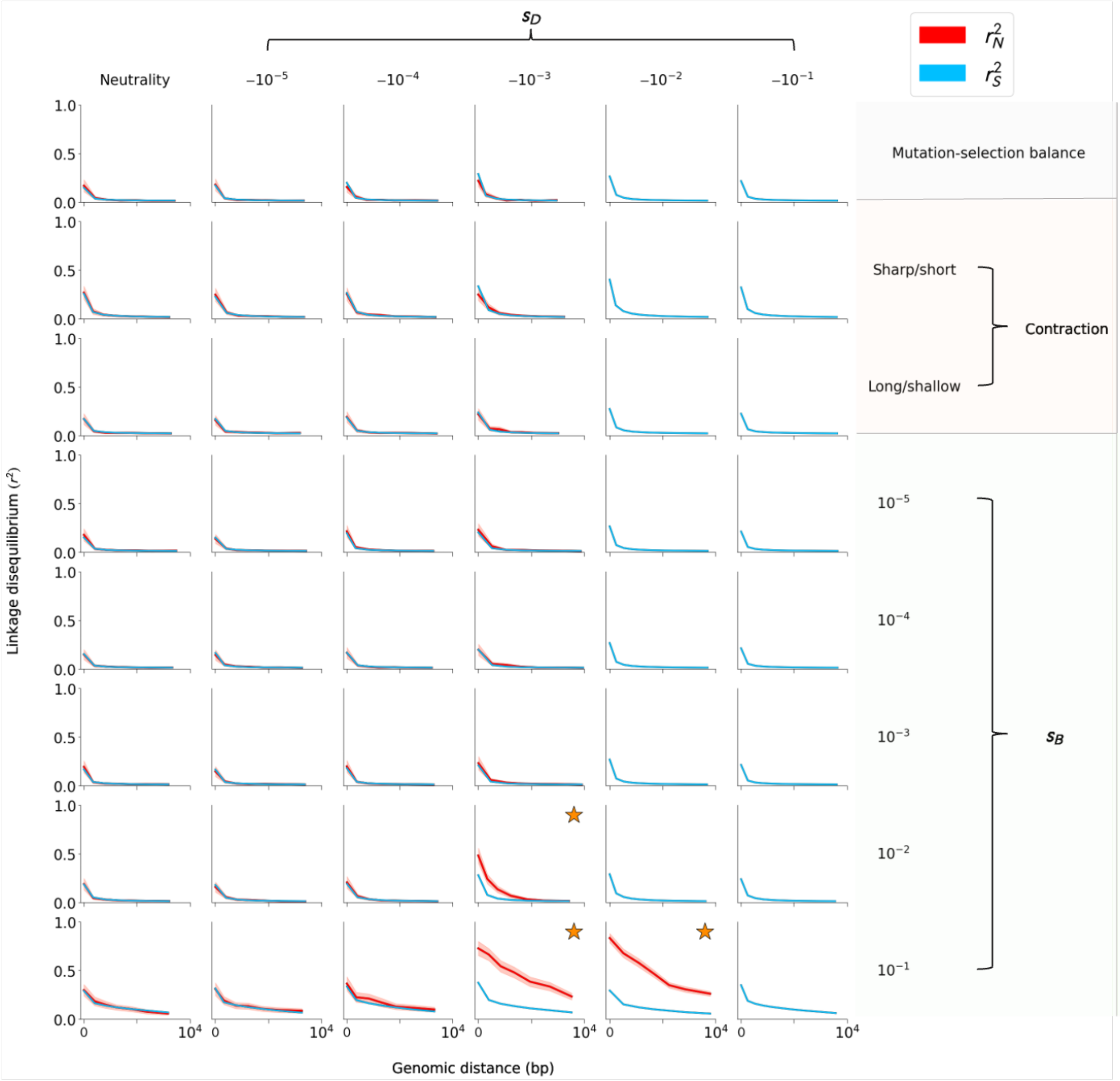

Figure S2: r_N^2 and r_S^2 measured among common variants in a range of simulated evolutionary scenarios, where $\rho = 10^{-6}$. Analogous to Figure **S1**, but for simulations performed with $\rho = 10^{-6}$ (i.e. $\rho/\mu = 1$). As expected, LD decays more rapidly in this regime than with $\rho/\mu = 10$. As with $\rho/\mu = 10$, AUC($r_N^2 - r_S^2$) was significantly greater than zero when $N_e s_D > 1$ and $s_B > s_D$.

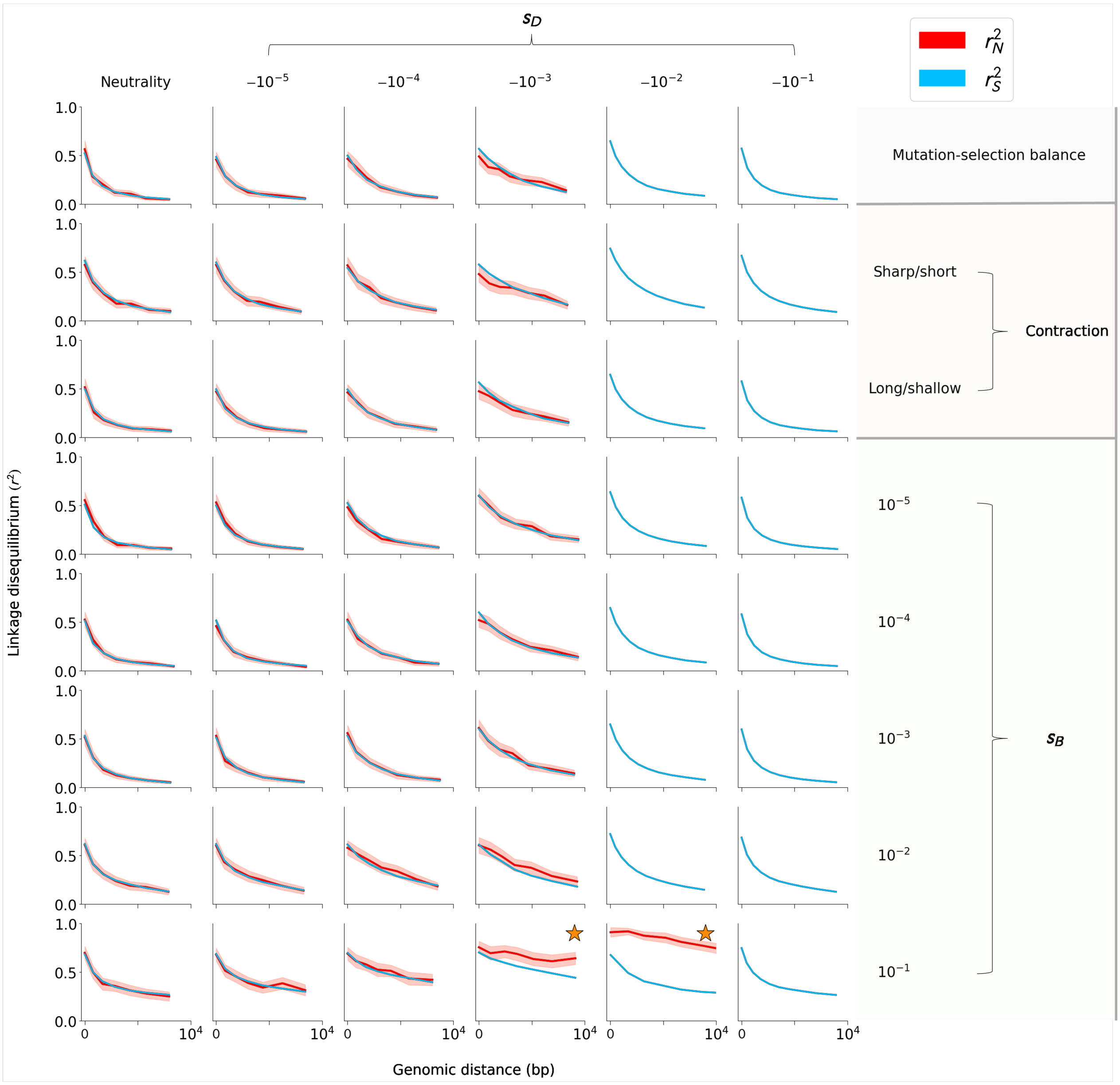

Figure S3: r_N^2 and r_S^2 measured among common variants in a range of simulated evolutionary scenarios, where $\rho = 10^{-7}$. Analogous to Figure **S1**, but for simulations performed with $\rho = 10^{-7}$ (i.e. $\rho/\mu = 10^{-1}$). As expected, LD decays much more slowly in this regime than with $\rho/\mu = 1$. Moreover, AUC($r_N^2 - r_S^2$) was significantly greater than zero only when $s_B = 10^{-1}$. While AUC($r_N^2 - r_S^2$) > 0 when $s_B = 10^{-2}$ and $s_D = -10^{-3}$, the difference was not significant.

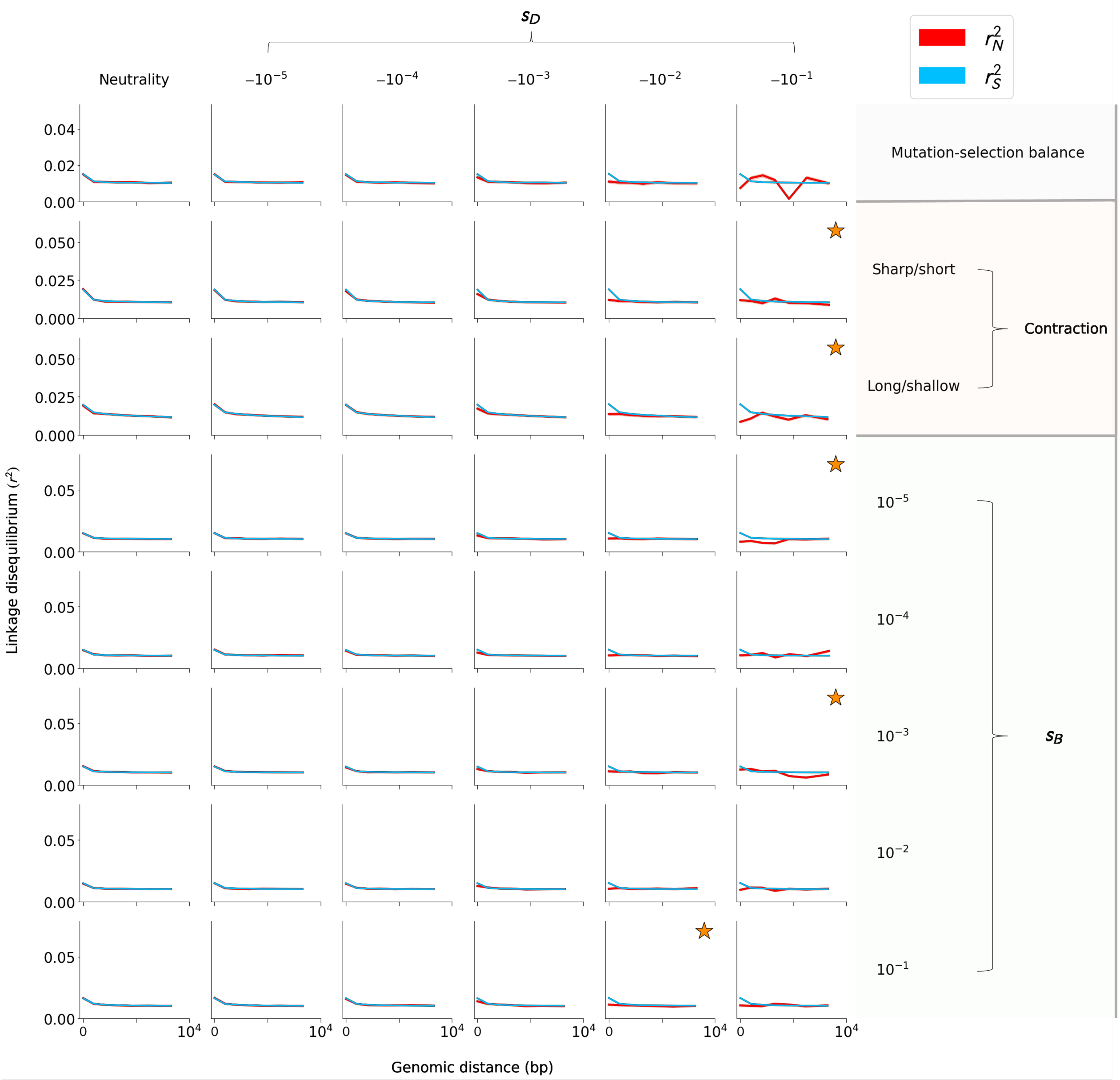

Figure S4: r_N^2 and r_S^2 measured among rare variants in a range of simulated evolutionary scenarios, where $\rho = 10^{-5}$. LD patterns among rare variants for the evolutionary scenarios discussed in the Main Text, with $\rho = 10^{-5}$ (i.e. $\rho/\mu = 10$). LD among both synonymous and non-synonymous variants decays extremely rapidly, and differences between r_N^2 and r_S^2 are typically only apparent over very short length scales.

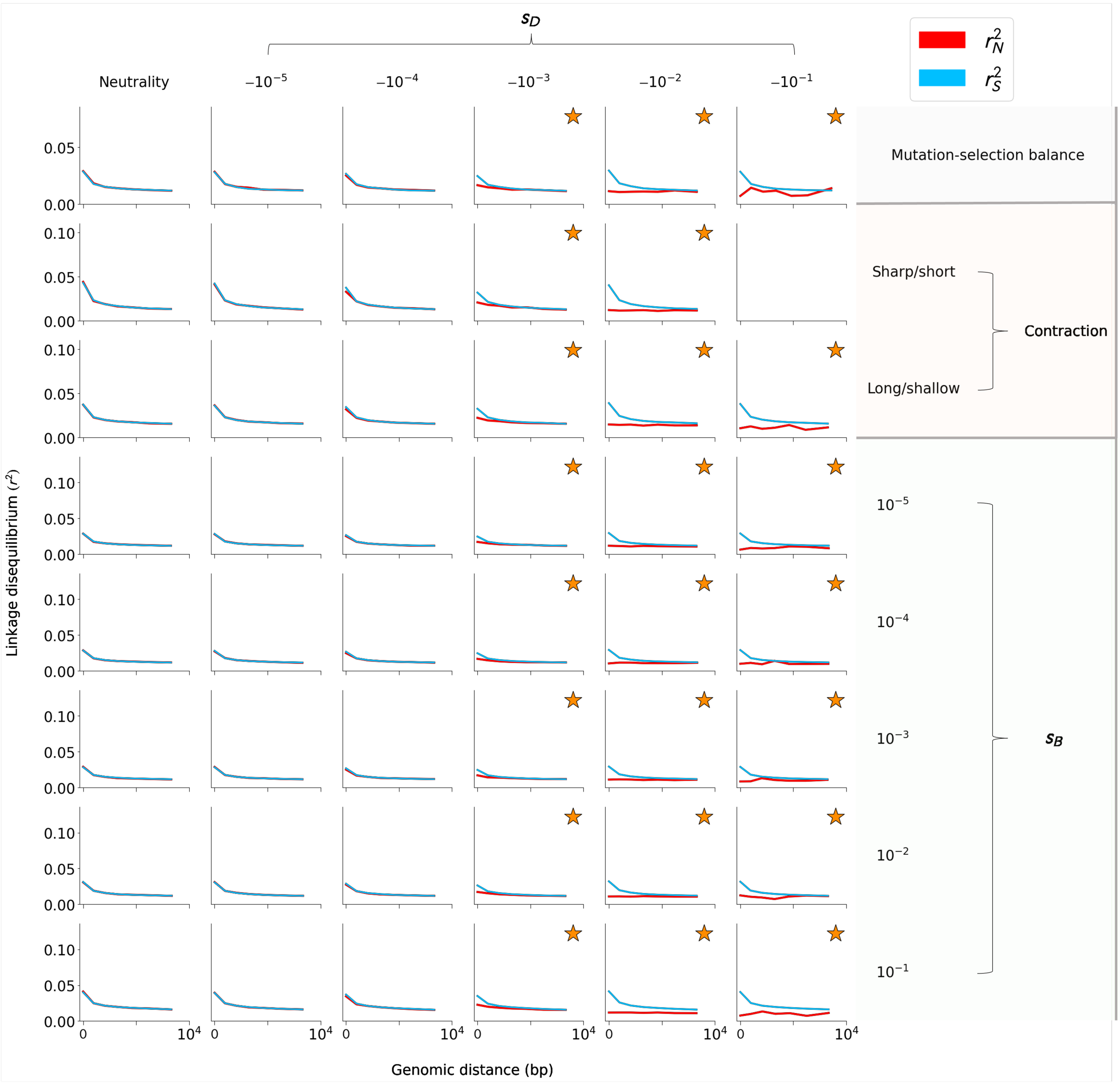

Figure S5: r_N^2 and r_S^2 measured among rare variants in a range of simulated evolutionary scenarios, where $\rho = 10^{-6}$. LD patterns among rare variants for the simulations discussed in the Main Text. AUC($r_N^2 - r_S^2$) is significantly less than zero in all simulations where $s_D > 1/N_e$ except for the short, sharp contraction with $s_D = -10^{-1}$, in which there were an insufficient number of both rare synonymous and non-synonymous variants to create LD curves.

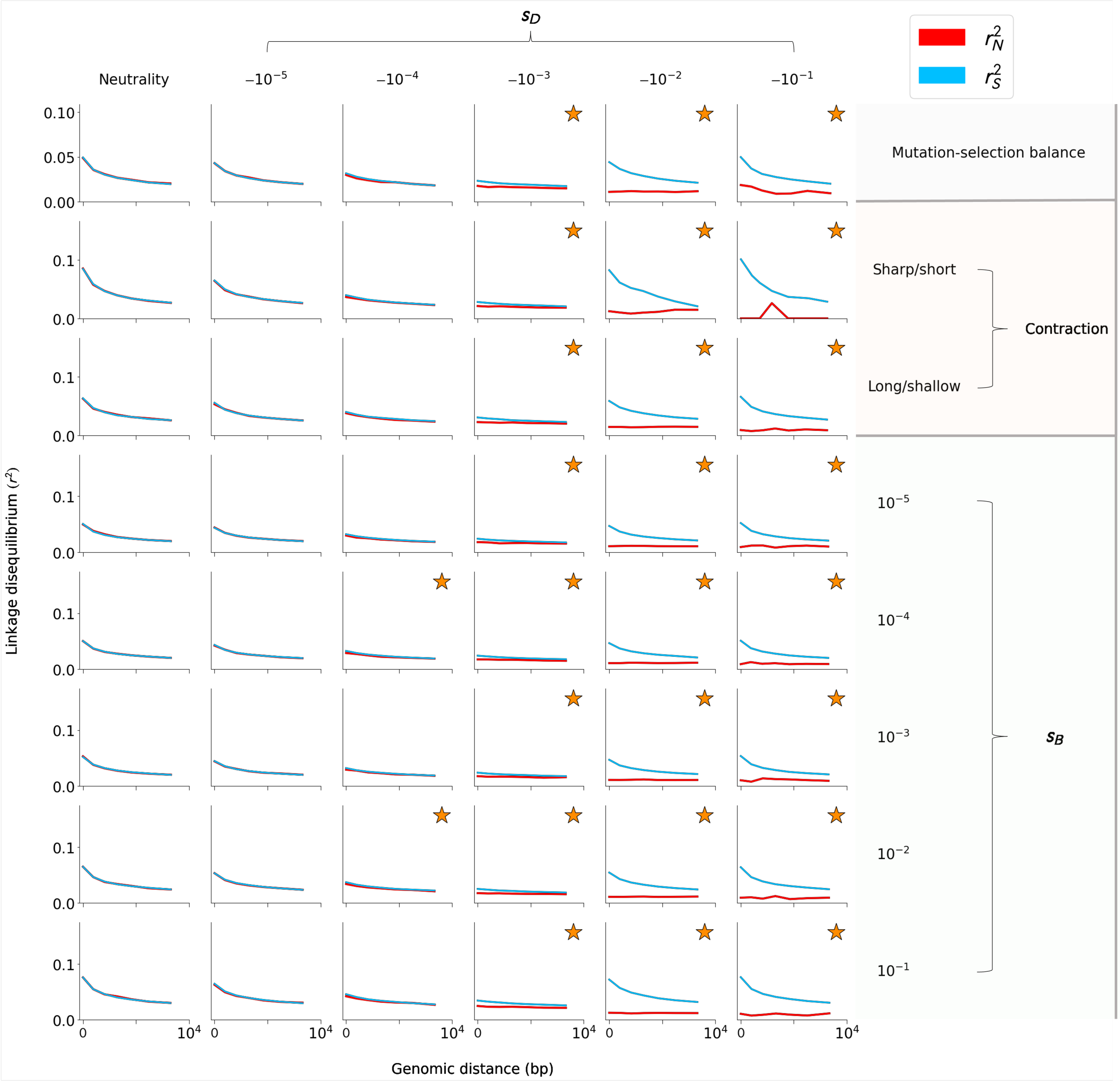

Figure S6: r_N^2 and r_S^2 measured among rare variants in a range of simulated evolutionary scenarios, where $\rho = 10^{-7}$. LD patterns among rare variants for the evolutionary scenarios discussed in the Main Text, with $\rho = 10^{-7}$ (i.e. $\rho/\mu = 10^{-1}$). AUC($r_N^2 - r_S^2$) is significantly less than zero in all simulations where $s_D > 1/N_e$, and in two instances also when $s_D = 1/N_e$.

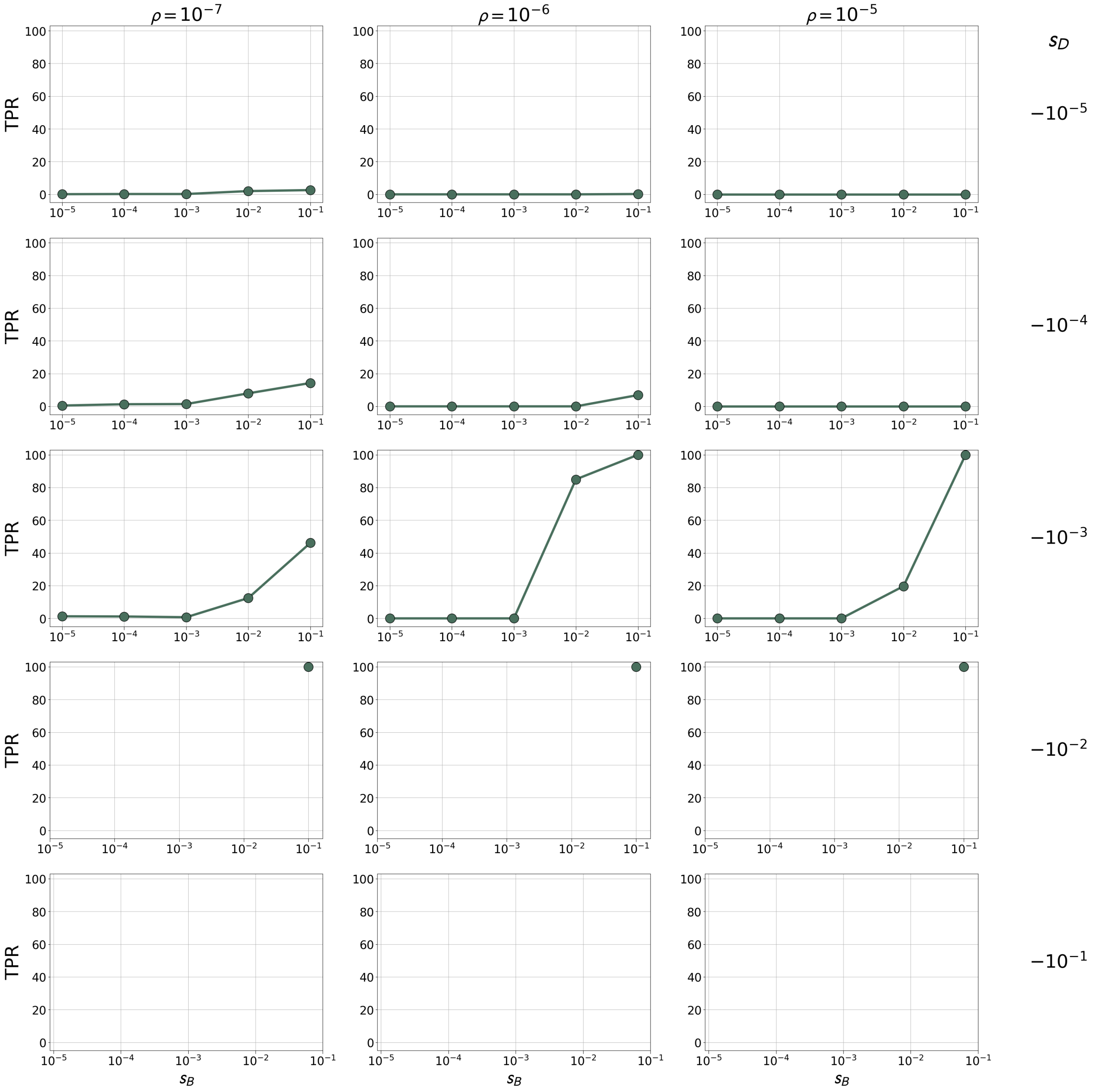

Figure S7: True positive rate of iLDS in simulated selective sweeps. To test the ability of iLDS to identify true instances of selection, we computed the statistic on our simulations of selective sweeps (Section 4.3). For each parameter combination (ρ, s_B, s_D) the true positive rate (TPR) for iLDS—i.e. the percentages of simulated populations for which iLDS was significant—is plotted. If an insufficient number of common non-synonymous variants were present in all simulations (as was the case whenever $s_D \geq s_B$), no TPR is plotted. iLDS was frequently significant when $N_e s_D > 1$ and $s_B > s_D$. When $s_D = -10^{-2}$, non-synonymous variants were only found at intermediate frequency if $s_B = 10^{-1}$, and iLDS correctly identified 100% of sweeps under these conditions.

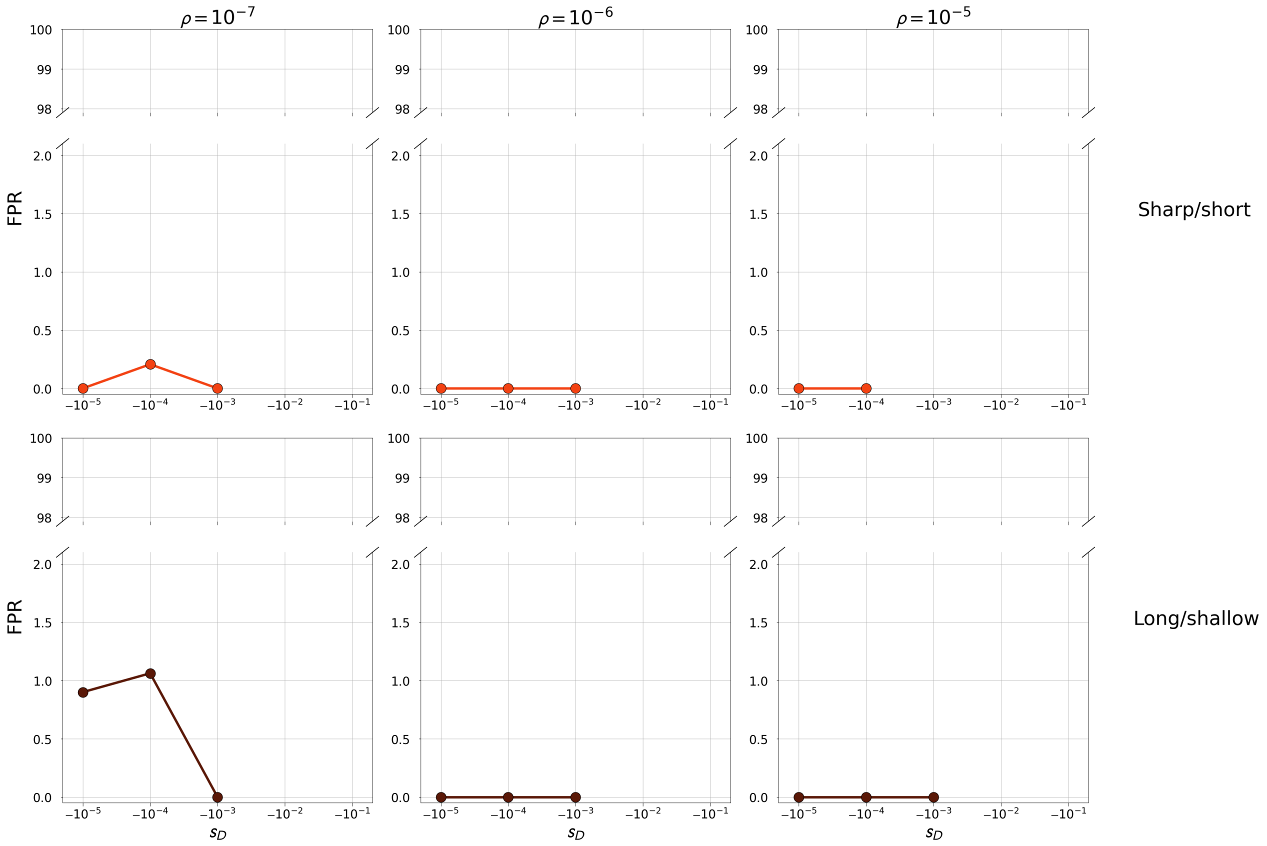

Figure S8: False positive rate of iLDS in simulated demographic contractions. To test iLDS's susceptibility to misidentifying demographic contractions as selective sweeps, we computed the statistic on our simulated demographic contractions (Section 4.3). For each contraction scenario (ρ, sharp/short or long/shallow) the false positive rate (FPR) for iLDS—i.e. the percentages of populations for which iLDS was significant—is plotted. If an insufficient number of common non-synonymous variants were present across all simulations (as was the case whenever s_D was large), no FPR is plotted. Note that the y-axis is broken at an FPR of 2%, and that no demographic scenario had an FPR exceeding $\approx 1\%$.

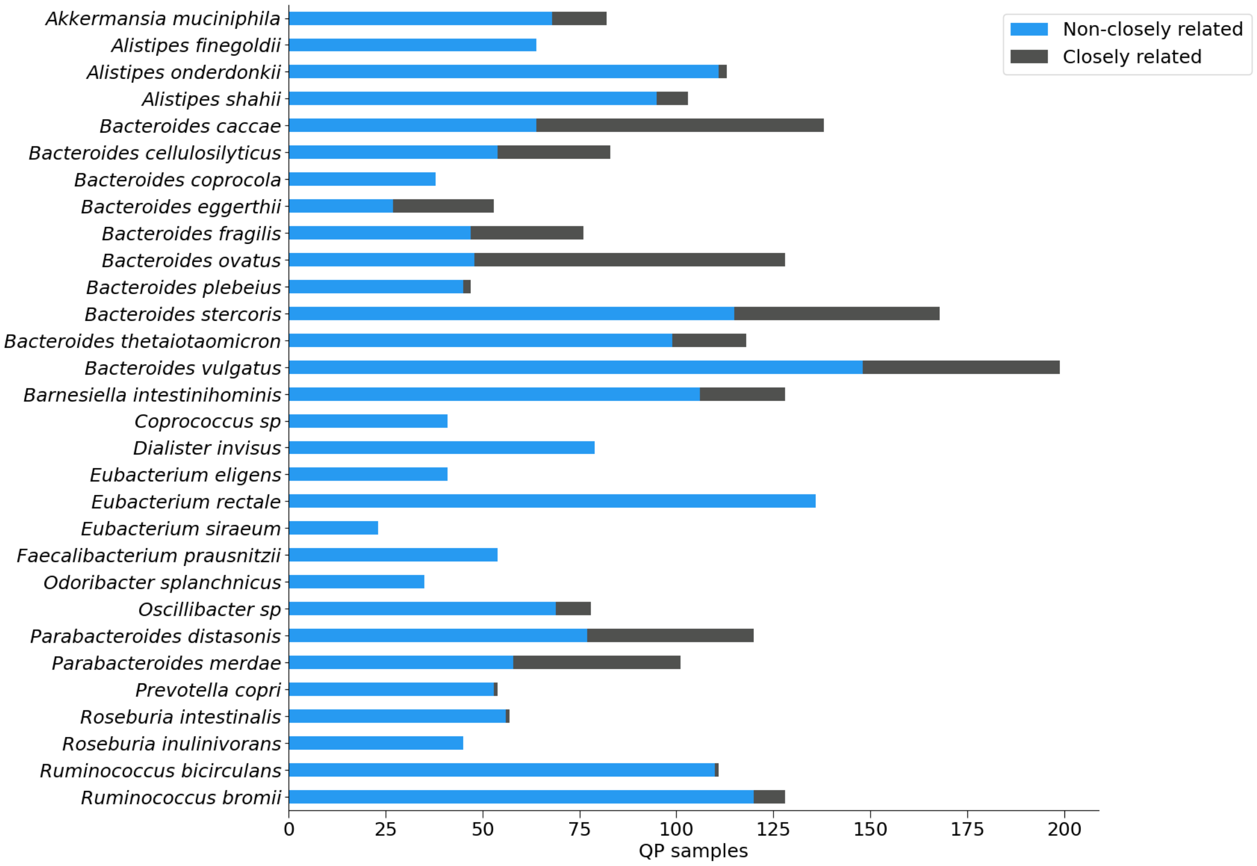

Figure S9: Number of QP samples by species. The number of QP samples (Supplementary Section 3.4) belonging to the largest clade for each of the 30 species analyzed. Samples are divided between closely related ($d < 5 \times 10^{-4}$) and non-closely related samples.

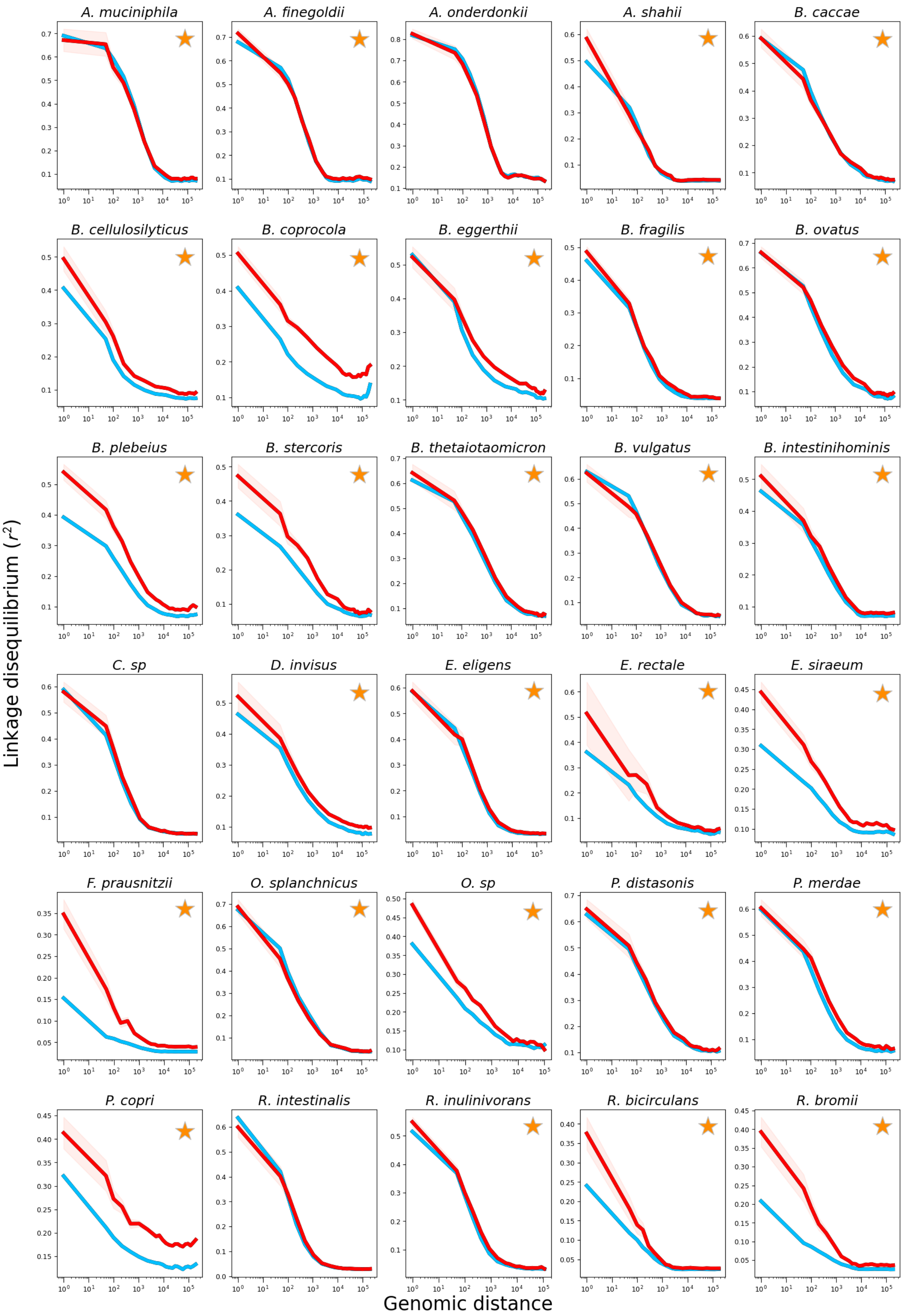

Figure S10: r_N^2 and r_S^2 measured in prevalent commensal gut microbiota for common variants. This figure is analogous to Figure 2A (top panel) in the Main Text. r_N^2 is shown in red, while r_S^2 is shown in blue. While $r_N^2 < r_S^2$ at certain distances in some species (e.g. at short distances in *Akkermansia municiphilia*, top left), overall r_N^2 typically exceeds r_S^2 for all species.

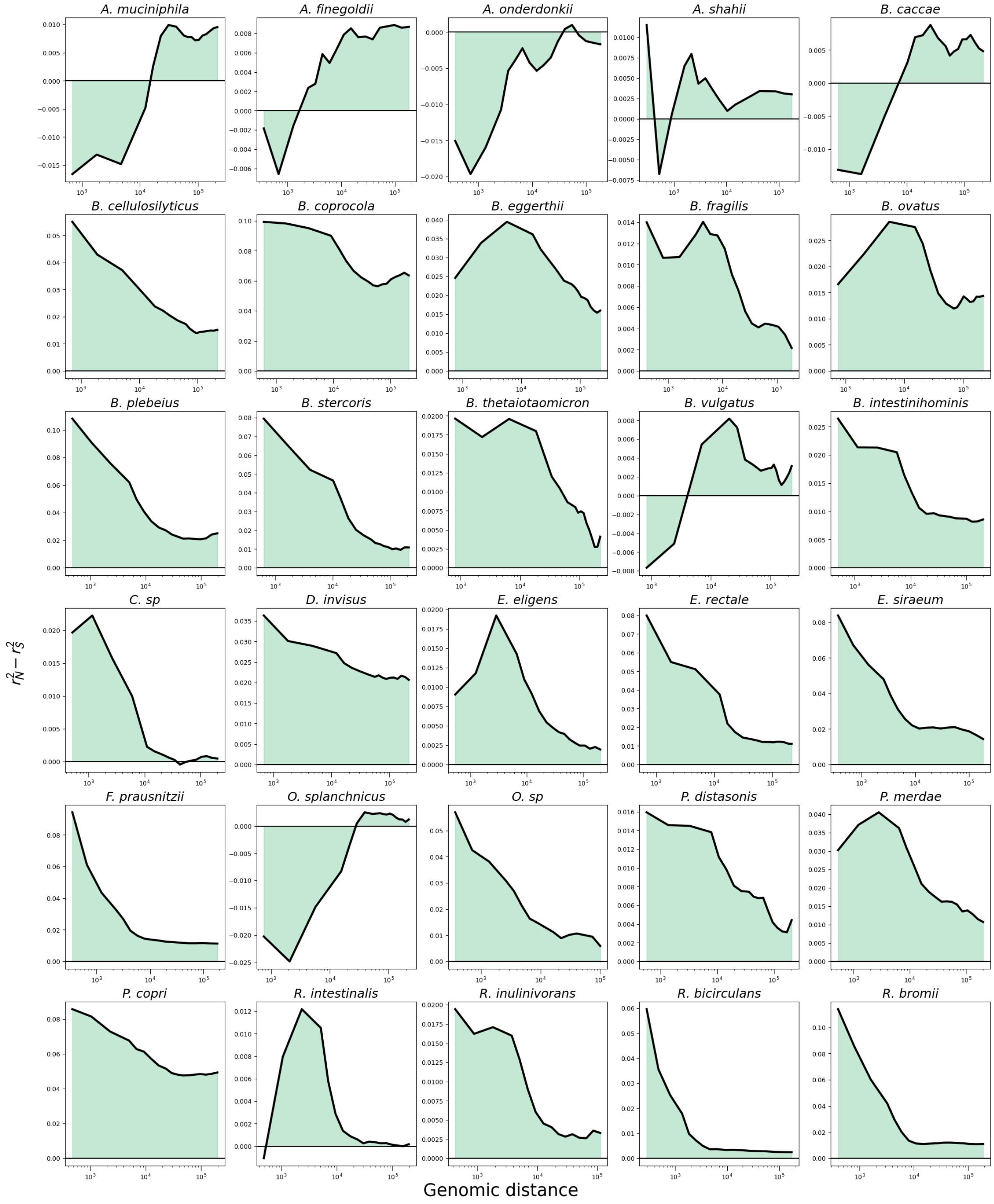

Figure S11: $(r_N^2 - r_S^2)$ **measured in prevalent commensal gut microbiota for common variants.** Here, the function $(r_N^2 - r_S^2)$ is plotted (black line), and AUC$(r_N^2 - r_S^2)$ is shown with the green shaded region. In a majority of species, $(r_N^2 - r_S^2)$ tends to decrease as a function of distance, or reach a maximum at some intermediate distance. A minority of species (such as *Akkermansia municiphilia*, top left) show an increase of $(r_N^2 - r_S^2)$ with distance.

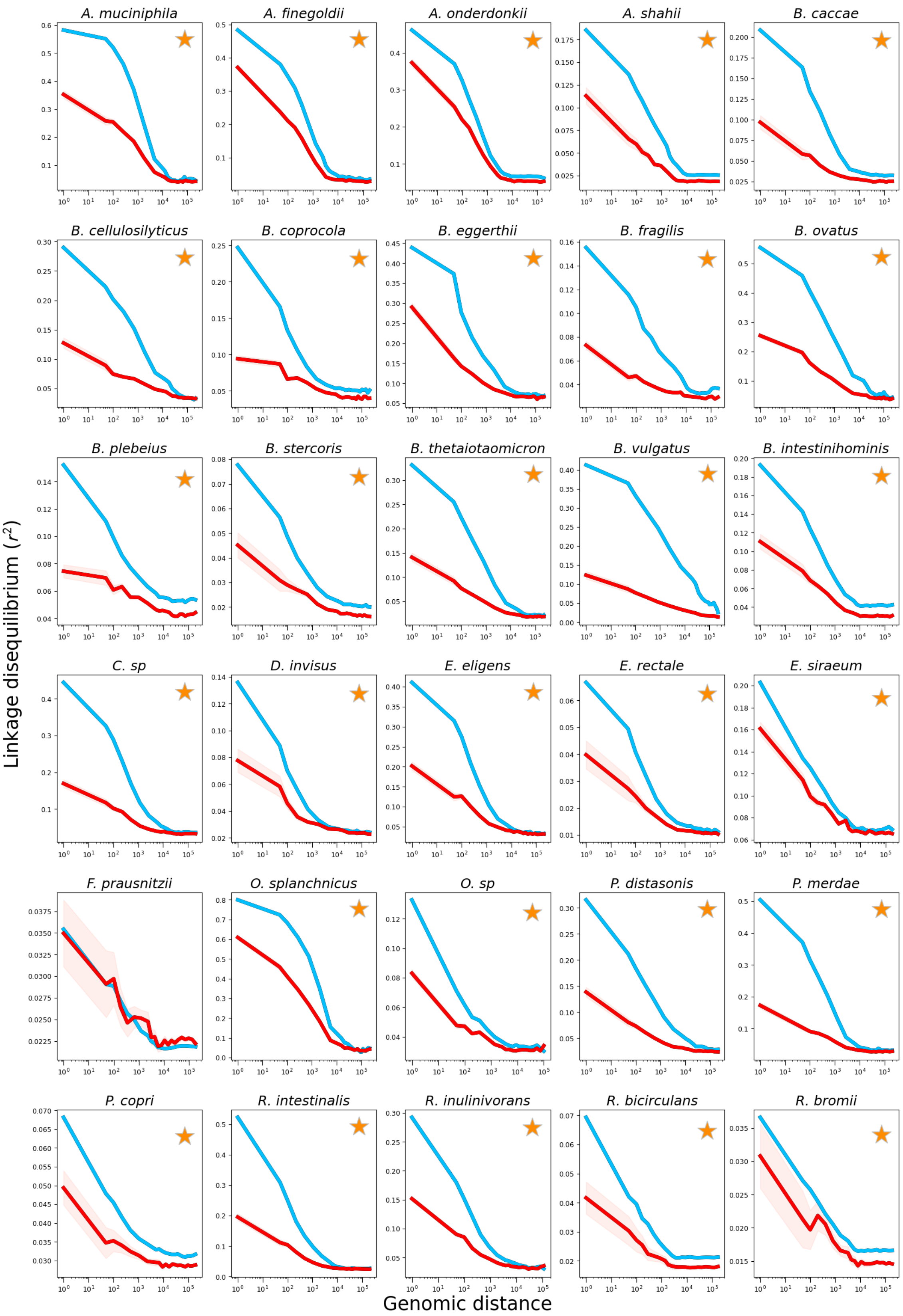

Figure S12: r_N^2 and r_S^2 measured in prevalent commensal gut microbiota for rare variants. This figure is analogous to Figure 2A (bottom panel) in the Main Text. r_N^2 is shown in red, while r_S^2 is shown in blue. r_S^2 is significantly elevated above r_N^2 in all but one species.

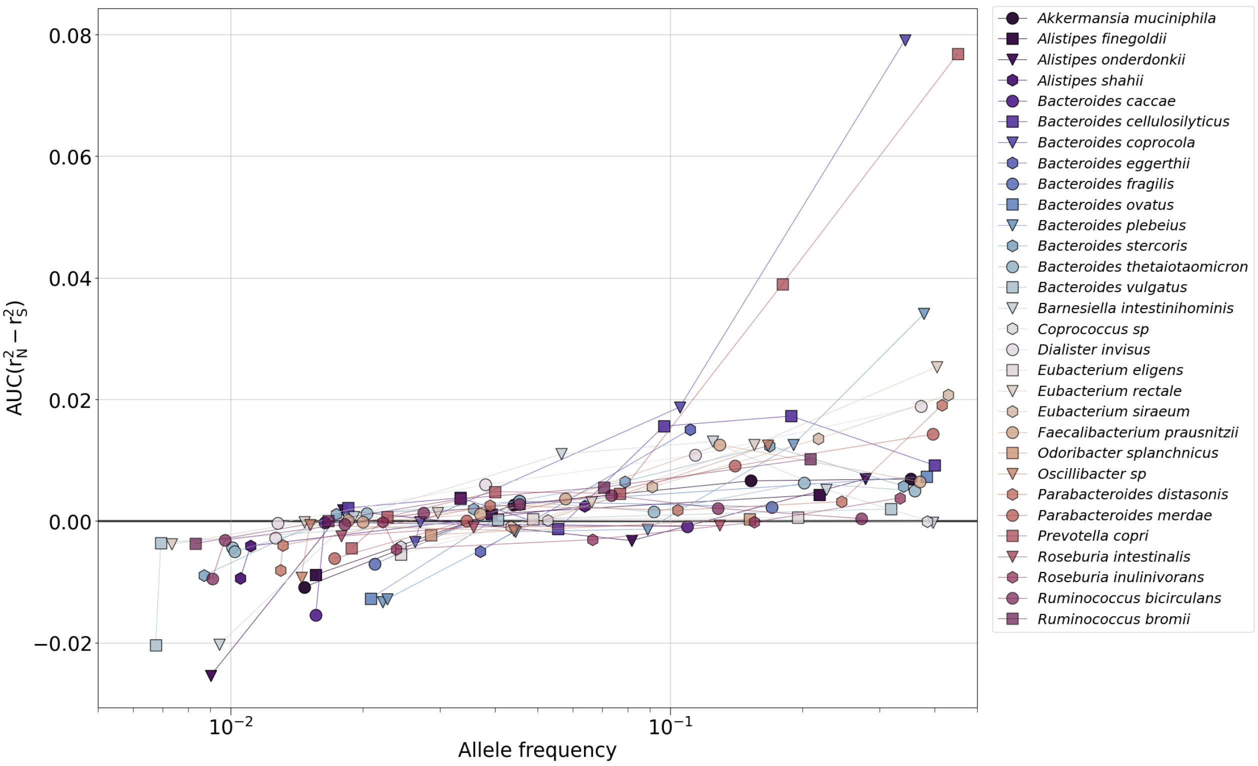

Figure S13: AUC($r_N^2 - r_S^2$) measured in prevalent commensal gut microbiota for varying allele frequencies. For each species, all variants were binned by allele frequency interval, and AUC($r_N^2 - r_S^2$) calculated for each bin. AUC($r_N^2 - r_S^2$) is universally an increasing function of allele frequency, with a transition between negative AUC($r_N^2 - r_S^2$) and positive AUC($r_N^2 - r_S^2$) typically occuring around $f \approx 0.05$.

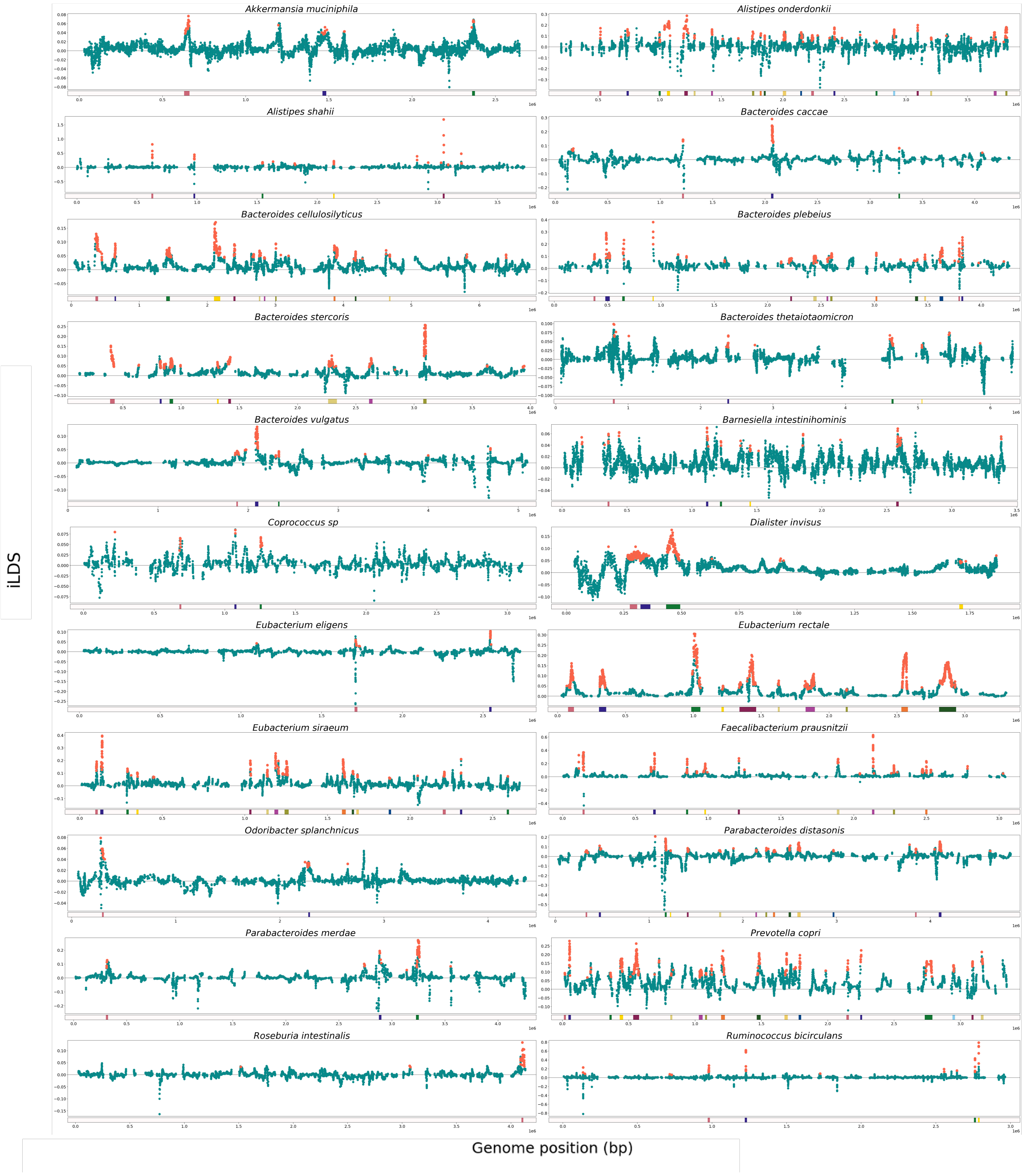

Figure S14: iLDS scans for all QP species analyzed not shown in Main Text. Analogous to Figure 3B, iLDS scans are shown here for the remaining 22 species in our dataset (QP metagenomes) with non-fragmented genomes. Significant windows are shown in orange, while non-significant windows are shown in green.

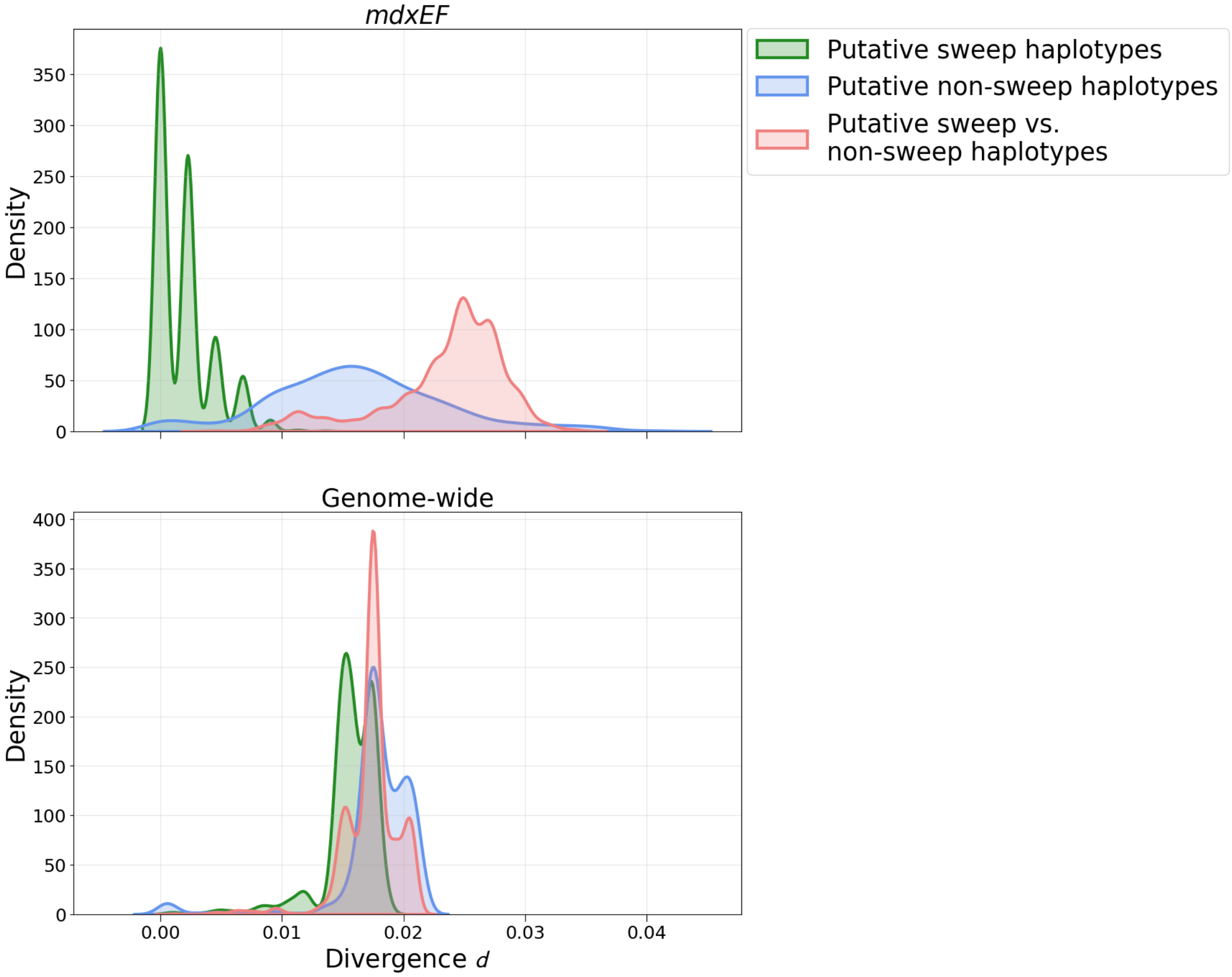

Figure S15: Genomic diveristy at the *mdxEF* locus versus genome-wide for the species *R. bromii*. For the species *R. bromii*, divergence d at 4D sites is shown at *mdxEF* (top) and genome-wide (bottom). The distribution of d among samples is shown for the putatively sweeping haplotype group in green (the 26 haplotype groups at the top of Main Text Figure 3), for the remaining haplotype groups in blue, and between these two groups in red. At *mdxEF*, the putatively sweeping haplotype groups have very low d relative to their genome-wide average, while the remaining haplotype groups have d comparable to their genome-wide averge (though with greater variance, as is expected given that d is calculated over far fewer sites). Moreover, the putatively sweeping haplotypes are far more genetically distinct from the remaining haplotypes at *mdxEF* than genome-wide.

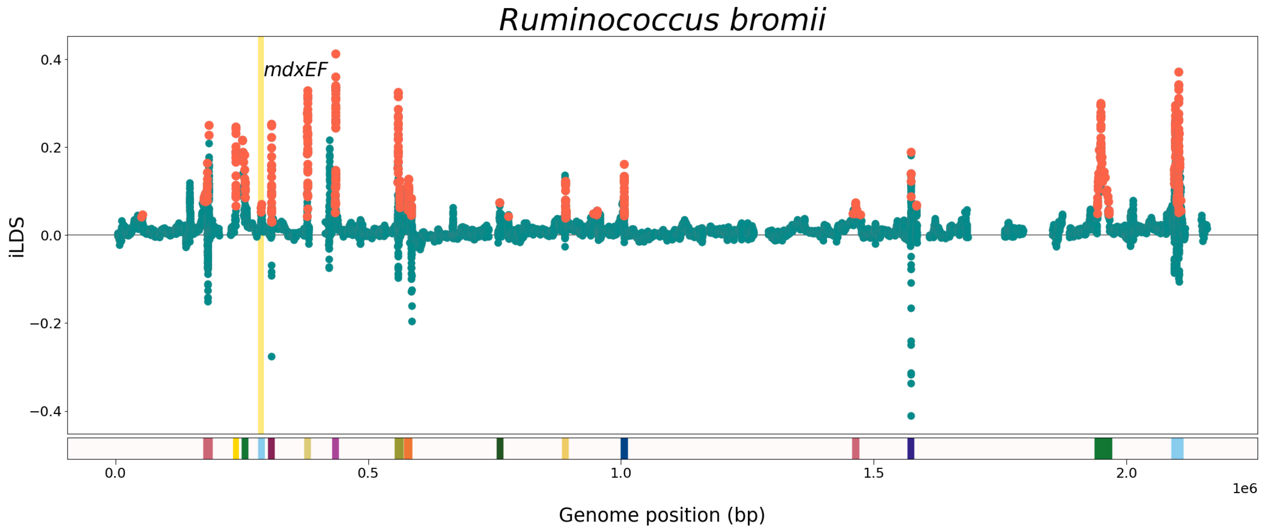

Figure S16: iLDS scan for *R. bromii*, UHGG. iLDS was run on 20 isolates of *R. bromii*. The location of the *mdxEF* genes, which lie in a peak, is highlighted (see Section 6 for further details on isolates and procedure for cross referencing peaks with the QP metagenome scan for the same species. Significant windows are shown in orange, while non-significant windows are shown in green. Locations of peaks are shown in the bar, at bottom.)

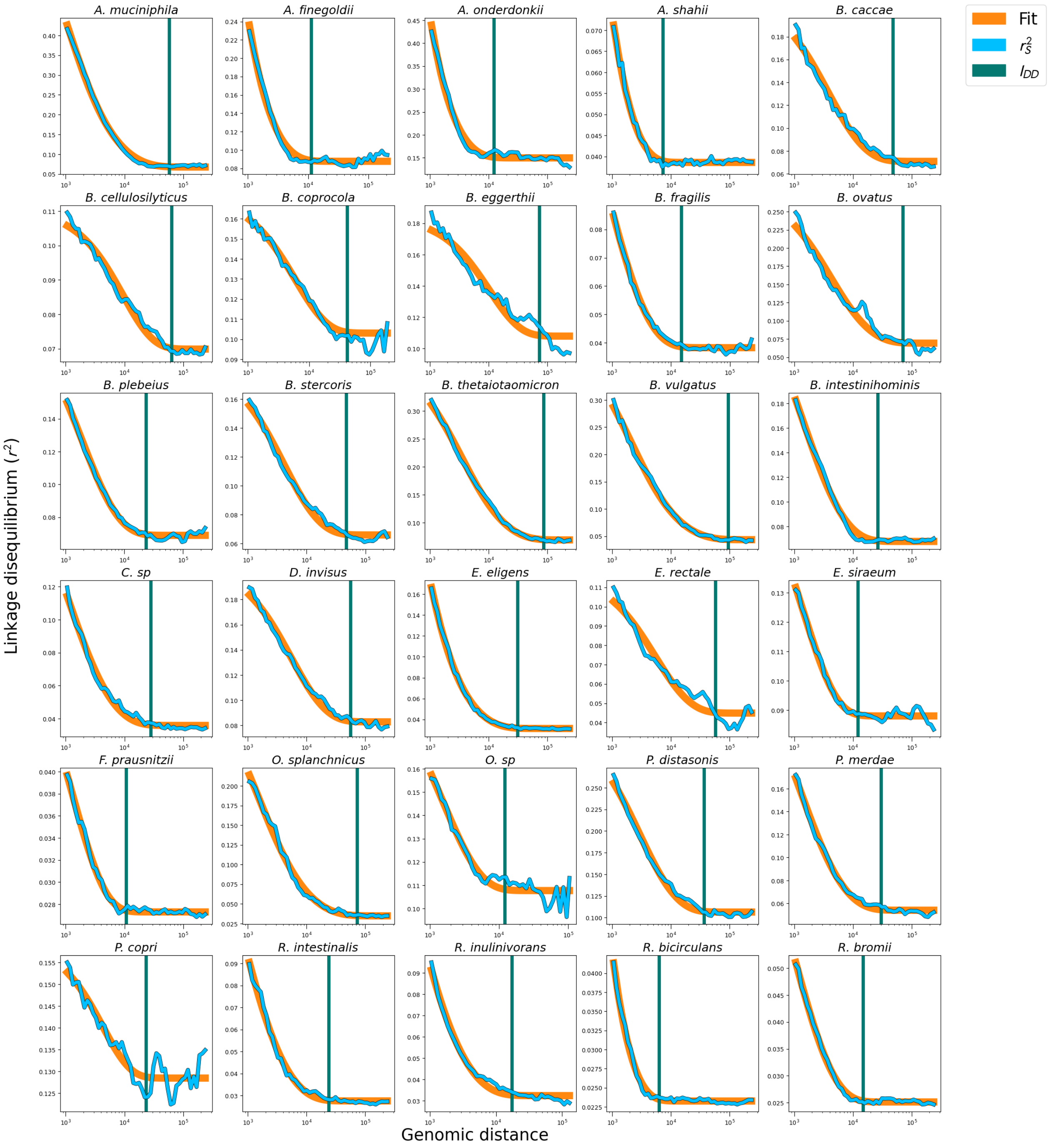

Figure S17: Estimates of l_{DD} for common commensal gut microbiota. Using the procedure outlined in Supplementary Section 4.1, we fit Equation S7 to r_S^2, and used the parameters of this fit to infer the decay distance l_{DD} (vertical lines). In general, r_S^2 appears to saturate at some long distance constant value in most species, and l_{DD} appears to approximate the length scale over which this saturation occurs.

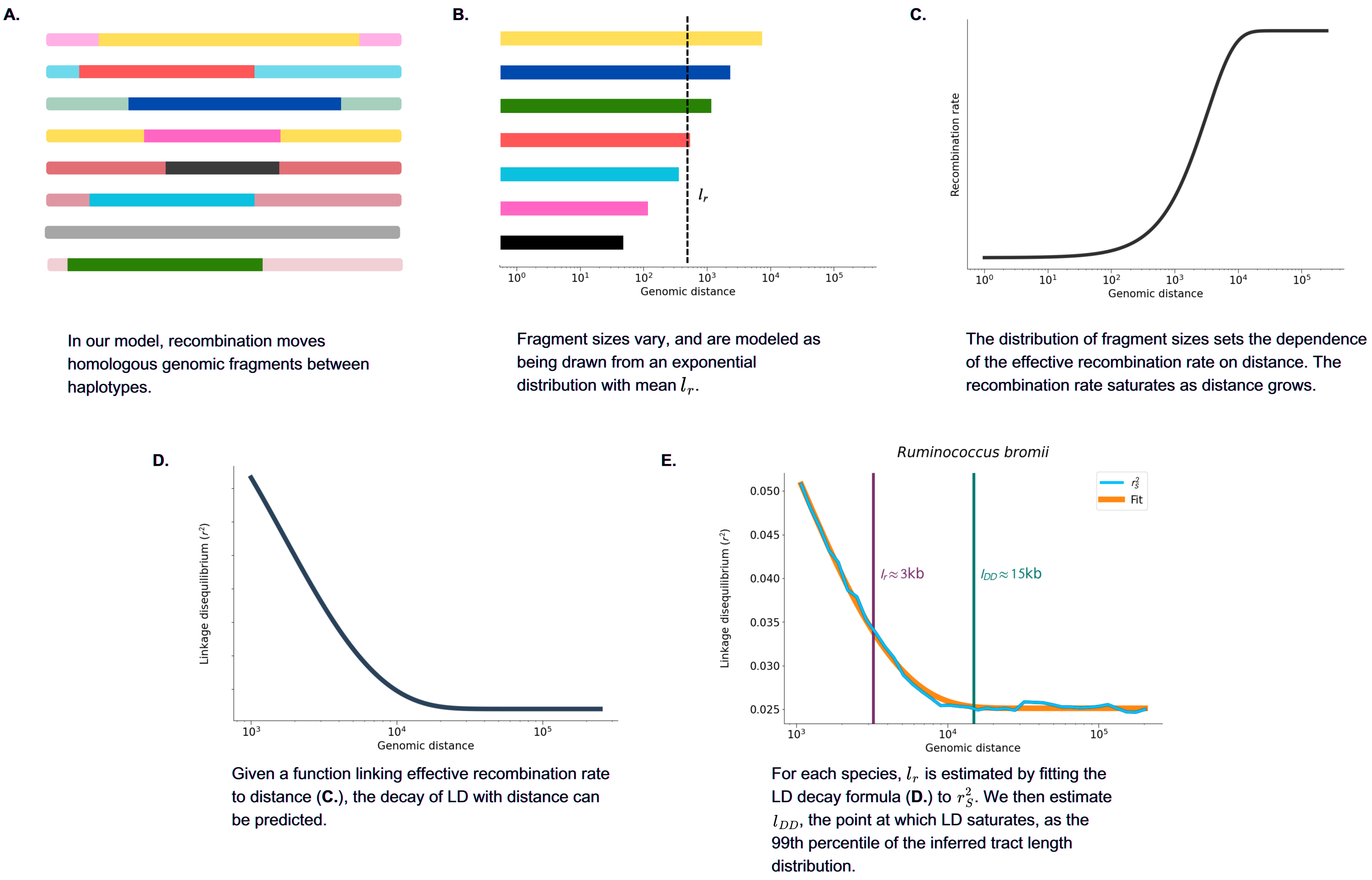

Figure S18: Schematic of method for inferring l_r and l_{DD}. Visual illustration of the procedure outlined in Supplementary Section 4.1, where Equation S7 is fit to r_S^2, and the parameters of this fit to infer the decay distance l_{DD} (in turn used to determine the window size for iLDS) in the microbial species examined here.

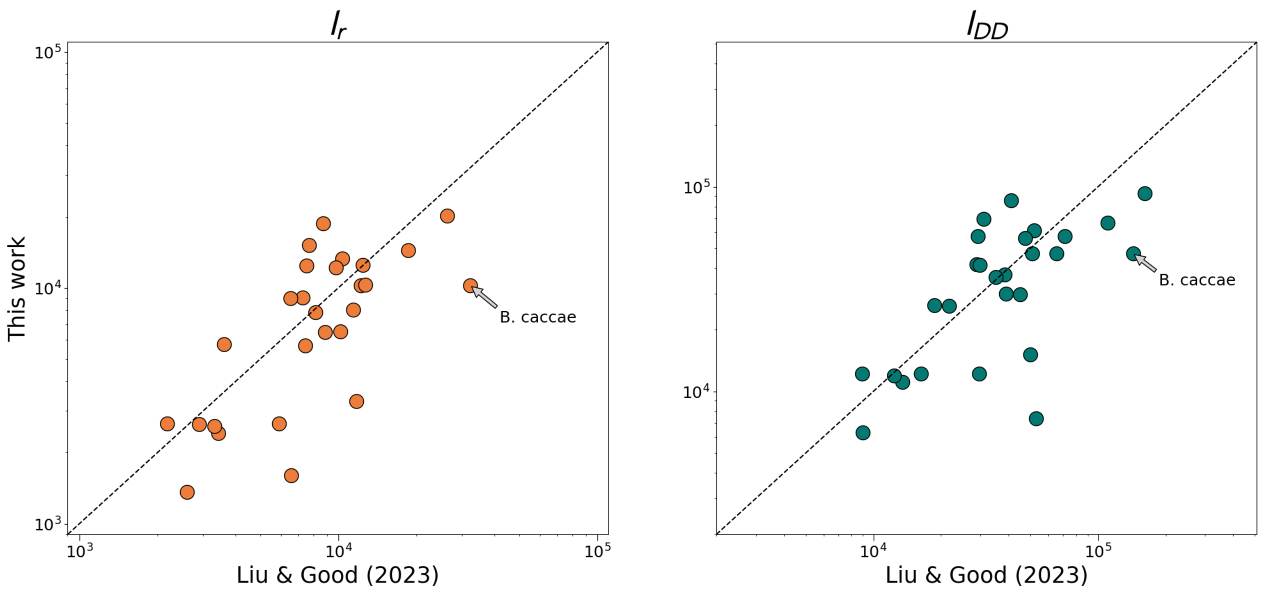

Figure S19: Comparison of l_r and l_{DD} estimates with Liu & Good (2023). We compared the values of l_r (left) and l_{DD} (right) obtained using the procedure outlined in Supplementary Section 4.1 with estimates of these values inferred from Supplementary data in Liu & Good (2023). The dashed line denotes the 1:1 line.

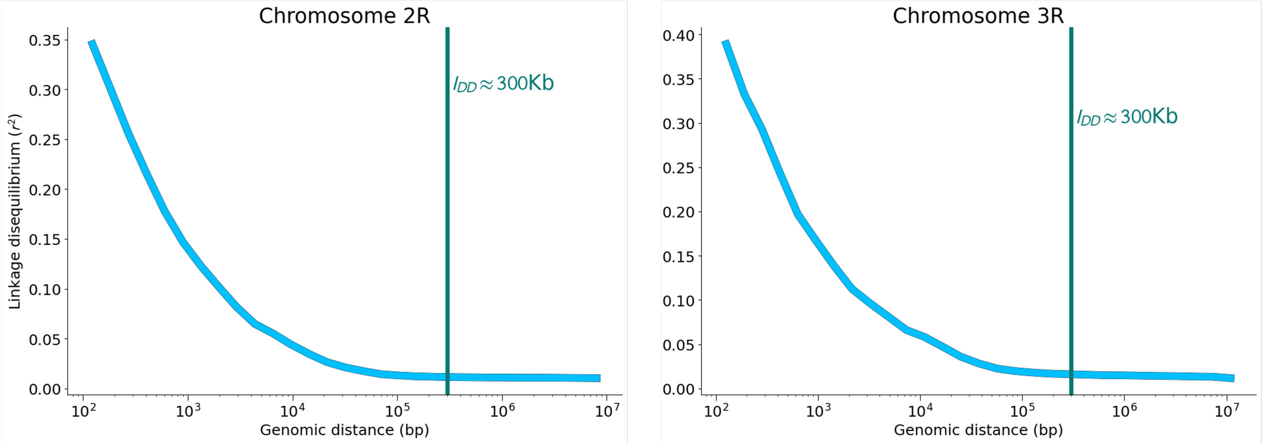

Figure S20: Decay of LD among common variants in *D. melanogaster*. Common variant r_S^2 curves and l_{DD} points used for iLDS scans for *D. melanogaster* chromosomes 2R and 3R. As recombination in *D. melanogaster* is governed by crossing-over rather than tract exchange, l_{DD} was estimated visually as the approximate point at which LD had fully decayed, rather than by using the scheme described in Section 4.1. As with the scans in bacteria, the window size in number of common non-synonymous SNPs was taken to be the median number of such SNPs necessary to cover l_{DD}.

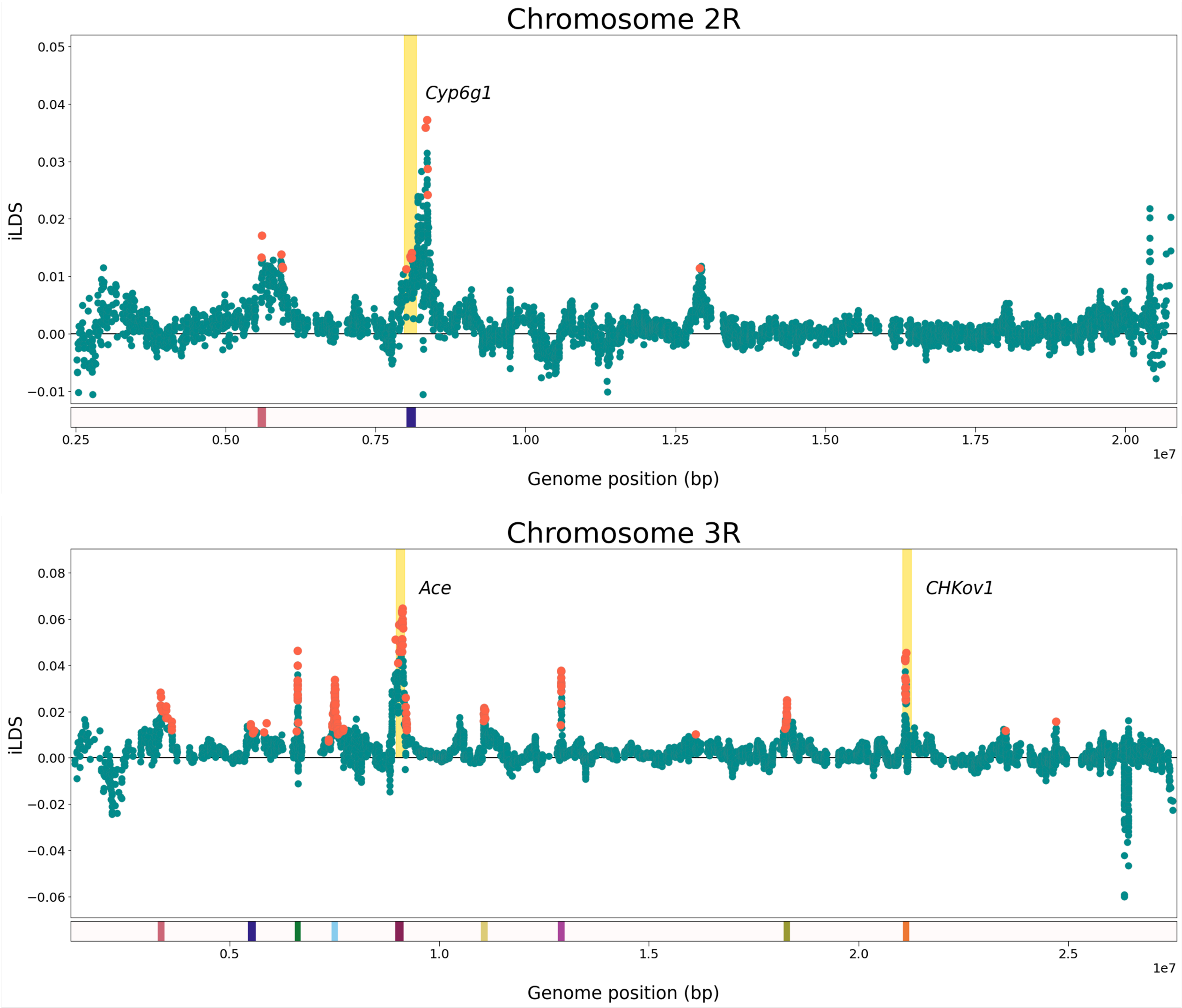

Figure S21: iLDS scans in *D. melanogaster*. iLDS scans for chromosomes 2R and 3R of *D. melanogaster*, where l_{DD} was set at 300Kb (Figure **S20**). The location of the yellow vertical line corresponds to the location of known selective sweeps at *Cyp6g1* (2R) and *Ace* and *Chkov1* (3R) plus 50kb to either side of the locations. Significant windows are shown in orange, while non-significant windows are shown in green. Locations of peaks are shown in the bar, at bottom.

1 LD

1.1 LD calculations

To calculate r^2 between two loci, we used the standard formula:

$$r^2 = \frac{(f_{AB} - f_A f_B)^2}{(f_A(1 - f_A) f_B(1 - f_B))} \tag{S1}$$

where f_A is the frequency of the minor allele at locus 1, f_B is the frequency at locus 2, and f_{AB} is the frequency of the AB haplotype. r^2 measurements were binned by genomic distance (bp) between the two loci, and mean r^2 within each bin was computed to determine decay in r^2 as a function of distance. We denote the location of the ith bin to be d_i, and the mean r^2 within this bin $\langle r_X^2(d_i) \rangle$.

1.2 AUC

To compute AUC(r_X^2)—the normalized area under the LD decay curve—we first approximate the total area under the curve using the trapezoidal rule and then divide by the total distance horizontal distance of the curve:

$$\text{AUC}(r_X^2) = \frac{1}{d_M - d_1} \sum_{i=1}^{M-1} \frac{\langle r_X^2(d_{i+1}) \rangle - \langle r_X^2(d_i) \rangle}{2} (d_{i+1} - d_i) \tag{S2}$$

where M is the total number of bins. The trapezoidal rule was implemented computationally with the trapz function in numpy [7].

1.3 LD confidence intervals

To quantify whether one LD curve is significantly elevated relative to another (e.g. r_N^2 versus r_S^2), we estimate confidence intervals (CIs) for the more elevated LD curve and assess if area under the other LD curve is lower than the area under the lower CI. Once LD measurements are binned by distance and the mean value ($\langle r^2(d_i) \rangle$) and standard deviation ($\sigma^2_{r^2(d_i)}$) computed for each bin d_i, we compute a confidence interval for the sample mean within each bin:

$$\text{CI}(\langle r^2(d_i) \rangle) = \langle r^2(d_i) \rangle \pm z^* \frac{\sigma^2_{r^2(d_i)}}{\sqrt{n_i}} \tag{S3}$$

where the z-score z^* sets the width of the interval used to assess significance, and n_i is the number of measurements in bin i. To determine if r_N^2 significantly exceeded r_S^2, we first computed a lower CI of r_N^2 (${r_N^2}^{(LCIB)}$) as follows:

$$r_N^2(d_i)^{(LCIB)} = r_N^2(d_i) - z^* \frac{\sigma^2_{r_N^2(d_i)}}{\sqrt{n_i}} \tag{S4}$$

then calculated AUC(${r_N^2}^{(LCIB)}$) and compared this quantity with AUC(r_S^2). If AUC(${r_N^2}^{(LCIB)} - r_S^2) > 0$ (that is, if the total area between the lower bound of r_N^2 and r_S^2 is positive), we considered r_N^2 to be significantly elevated above r_S^2. When determining if r_N^2 exceeded r_S^2 at the whole-genome level (Main Text Figure 2B), significance was assessed at the $\alpha = 99.9\%$ level ($z^* = 3.291$). In essence, we assess whether r_N^2 overall is greater than r_S^2, on average, taking into account the variability of r_N^2 at each distance. It is important to note that in order for r_N^2 to significantly exceed r_S^2, it is neither necessary nor sufficient that r_N^2 be greater than r_S^2 in all bins. For rare variants, purifying selection is expected to decrease linkage among non-synonymous variants relative to synonymous ones, and significance is therefore assessed with respect to the upper bound of r_N^2 (i.e. AUC(${r_N^2}^{(UCIB)}$) - AUC(r_S^2) < 0.)

To ensure that all species had comparably sized confidence intervals surrounding r_N^2 in our genome-wide assessment of significance, we downsampled the number of non-synonymous LD measurements to 1,000,000 for common variants (roughly 1,500 $\times$ 1,500 pairwise measurements) and 25,000,000 (roughly 7,000 $\times$ 7,000) for rare variants. Additionally, as the circular genome size in the species studied here varies, we only considered LD measurements of variants lying on the same contig and within 250Kb of one another. LD was binned in 20 logarithmically spaced bins, with short distance begins at 0 and 50bp.

2 Simulations

To evaluate the conditions under which linkage between non-synonymous variants might be elevated relative to linkage between synonymous variants, we performed evolutionary simulations in SLiM 4.0 [8] using custom recipes.

While the species examined in the empirical portion of this study are haploid (with the exception of *D. melanogaster*, Section 7 below), simulations were performed with diploids. Diploid evolution was chosen as a simulation paradigm for two reasons. First, diploid simulations in SLiM employ a standard Wright-Fisher models of evolution, aiding in the interpretability of the simulation results. Second, we aimed to maximize computational efficiency for the large number of simulations we performed (4500 burn-in simulations, $135,000$ sweeps, $32,500$ contractions) with population sizes of $N = 10^4$.

While haploid and diploid evolution differ in important ways, simulations were tailored to minimize the effect of these differences, while still retaining the performance advantages offered by simulations of diploidy in SLiM. First, all mutations were assigned a dominance coefficient of 1, making them fully visible to selection regardless of the presence or absence of any mutation on the opposite chromosome. In particular, this means that deleterious variants are always exposed to selection, as is the case with haploids. Second, while the crossing-over recombination typical of diploids differs from the homologous donation of tracts typical of bacterial evolution (at least in the core genome [9, 10]), for our purposes these differences manifest primarily in the efficiency with which genomic elements at large distances can be unlinked. Variants which are physically close to one another relative to the average recombined tract length l_r will be affected identically by crossing-over versus homologous recombination (see Section 4.1). As individuals in our simulated populations have chromosomes of length 10Kb, the decay of linkage should resemble that of a bacterial species with a tract length considerably larger than 10Kb, such as *B. vulgatus* ($l_r = 23$Kb).

To calculate LD, we first sampled 100 individuals from each simulated population. As with our genome-wide assessments, we next sampled a constant number of LD measurements between non-synonymous variants in our simulations to ensure that confidence intervals around r_N^2 were comparable across evolutionary scenarios. Specifically, for common variants, we sampled 1250 LD measurements between non-synonymous variants with replacement, for an average of one measurement per simulation for sweeps and contractions. Among rare variants, we sampled 1,000,000 LD measurements. We found, in general, that rare variant LD was more variable (less smooth decay, wider confidence intervals) than common variant LD for a given number of LD measurements,

likely reflecting the more limited range of potential LD values that can be taken on between pairs of rare variants. Pairs of singletons, for instance, may only take on r^2 values of zero or one.

If fewer than 100 LD measurements were available for a given parameter combination and mutation type, we did not calculate an LD decay curve (e.g. among non-synonymous variants for $s_B = 10^{-5}, s_D = 10^{-1}$, where the adaptive variant was always the only intermediate frequency non-synonymous variant following the sweep.) Following sampling, r_N^2 and r_S^2 were calculated using 8 bins each containing an equal number of non-synonymous variants. Significance was assessed in these simulations at the $\alpha = 99.9\%$ level, again mirroring the genome-wide measurements.

2.1 Burn-in/mutation-selection balance

In each replicate population of size of $N = 10^4$ individuals, burn-in proceeded for $10N$ generations, allowing the population to reach mutation-selection balance. Populations were constituted of individuals with diploid genomes of size $L = 10^4$ bp. Within each simulation, s_D was held constant at all non-synonymous sites, and between simulations, s_D was varied from a nearly-neutral regime ($N_e s_D << 1$: $s_D = 0, -10^{-5}, -10^{-4}$) through a weakly deleterious regime ($N_e s_D \approx 1$: $s_D = -10^{-3}$ to a strongly deleterious regime ($N_e s_D >> 1$: $s_D = \{-10^{-2}, -10^{-1}\}$). Following the degeneracy of the codon table, we assumed a ratio of 2.31 non-synonymous sites per synonymous site in our simulations [11], with a per-basepair mutation rate $\mu = 10^{-6}$. Finally, the scaled recombination rate ρ/μ—i.e. the quotient of the recombination and mutation rates—was varied from 0.1 to 1 to 10 ($\rho = 10^{-7}, 10^{-6}, 10^{-5}$). For each parameter combination, 250 replicate populations were simulated.

2.2 Demographic contractions

To simulate demographic contractions, we began by first reading in a burn-in population with the desired s_D. In short, sharp contractions, the population size was reduced from $N = 10^4$ to $N = 10^3$, a 90% reduction, and the simulation was allowed to proceed for 20 generations. In the long, shallow contractions, the population size was reduced from $N = 10^4$ to $N = 5 \times 10^3$, a 50% reduction, and the simulation was then allowed to proceed for 5000 generations. For each burn-in population, five replicate contractions were performed, yielding a total of 1250 contraction simulations for each parameter combination.

2.3 Partial sweep

To simulate partial sweeps, we began by first reading in a burn-in population with the desired s_D, and then introducing a novel non-synonymous mutation at position zero on the chromosome. For each burn-in population, five replicate sweeps were performed, yielding a total of 1250 simulations per (ρ/μ, s_B, s_D) combination. Simulations proceeded until the focal, adaptive mutation reached 50% frequency, and were restarted from the burn-in state if the focal variant was lost from the population.

3 Metagenomic pipeline

3.1 Overview

In this work, we identify signatures of selection primarily in haplotypes obtained from publicly available fecal shotgun metagenomes of healthy Western adults. While such short read data can yield very accurate estimates of intra-sample allele frequencies, it inherently fragments the relatively long-range linkage patterns which are of interest to us. To be able to measure linkage disequilibrium on length scales exceeding that of the length of a short read, we first map reads to reference genomes, and then identify particular sample/species combinations in which a dominant haplotype can be extracted with high confidence and minimal probability of error (Section 3.4). Below, we describe the pipeline used to reconstruct these haplotypes from this metagenomic data.

3.2 Data

The raw sequencing reads for the 1,013 metagenomic samples used in these study were collected from 693 unique individuals (though only a single sample was used per person), including 250 individuals from the Human Microbiome Project (HMP) [12], a further 250 from Xie *et al* (2016) [3], 185 from Qin *et al* (2012) [4], and 8 from Korpela *et al* (2018) [5]. Metadata for each sample is available in Table S1.

3.3 Estimation of species, gene, and SNV content of shotgun metagenomic samples

Species abundances, gene, and SNV content for each metagenomic shotgun sample were previously quantified in Garud & Good (2019) [1] using MIDAS (Metagenomic Intra-Species Diversity Analysis System, version 1.2, downloaded on November 21, 2016) [13]. Here we briefly summarize again the steps followed to obtain the data relevant for this work.

3.3.1 Estimation of species content

Species abundances for each sample were estimated by mapping reads to a set of single copy marker genes belonging to the 5,952 species in the MIDAS database. A species was considered present if it had an average marker gene coverage ≥ 3. Since some hosts from the Human Microbiome Project dataset were sampled over multiple timepoints, we next determined a single reference database for

each host by including all species present at one or more timepoints with coverage $\geq$3. In doing so, we were as inclusive as possible of any species present at any time point to avoid ‘read donating’ to species that may resemble true species that are present, but also selective enough to prevent ‘read stealing’ from a species truly present.

3.3.2 Estimation of CNV content

CNV content was next estimated by mapping reads to the pangenome for each species in each per-host reference database using Bowtie 2 ([14]) with default MIDAS settings (local alignment, MAPID$\geq$94.0%, READQ$\geq$20, and ALN_COV$\geq$0.75). MIDAS estimates the copy number of any gene (c) as the ratio between its coverage and the median single marker gene coverage.

For each species, we identified “core genes”, defined as genes present in at least 90% of samples belonging to the largest clade (described in the Main Text and in Garud & Good (2019) [1]). Copy number values were used to estimate the prevalence of genes across hosts, defined as the fraction of samples with copy number $c \leq 3$ and $c \geq 0.3$ (conditional on the mean single gene marker coverage being $\geq 5\times$). In our calculation of LD, as well as for any iLDS scans, we only considered core genes. Since genes that are shared across species boundaries can result in read ‘stealing’ and ‘donating’, we excluded those genes part of a ‘blacklist’ of shared genes that we previously inferred [1]. In addition, since some genes may be absent from the MIDAS database, we also excluded genes with $c \geq 3$ in at least one sample in our cohort as in Garud & Good (2019) [1], to avoid examining genes that may have high copy number due to their being present in multiple species.

3.3.3 Estimation of SNV content

Next, to estimate SNV content, reads were mapped to a single reference genome per species previously selected in the default MIDAS software. Read mapping was performed with Bowtie 2 ([14]) using default MIDAS thresholds (global alignment, MAPID$\geq$94.0%, READQ$\geq$20, ALN_COV$\geq$0.75, and MAPQ$\geq$20). As per the defaults in MIDAS, species for a given sample were excluded from further analysis if $\leq$ 40% of their genome had any coverage or if they had median read coverage $\bar{D} < 5$ at protein coding sites with nonzero coverage. To further avoid read stealing and donating, sites were masked in a given sample if $D < \bar{D}/3$ or $D > 3\bar{D}$, as these sites harbor coverage anomalously low or high compared to the genome-wide average coverage $\bar{D}$. An additional coverage threshold requirement of 20 reads/site was imposed for calling SNVs for analyses below.

3.4 Quasi-phasing

Once SNVs had been determined within each sample, we obtained "quasi-phased" (QP) haplotypes from individual hosts following the procedure originally outlined in Garud & Good *et al.* (2019) [1]. Briefly, quasi-phasing estimates one of the dominant haplotypes for a given species in samples for which that species has high coverage ($\bar{D} > 20$) and relatively few intermediate frequency variants, allowing each allele to be confidently assigned to a single haplotype with low (and bounded) probability of polarization errors due to sampling noise. As our paper and Garud & Good (2019) analyze the same data, we leveraged the same sample x species classified as quasi-phaseable in that work. Any sites with intermediate frequency ($0.2 < f < 0.8$) polymorphism in the sample were treated as missing data, and the dominant major alleles ($f \geq 0.8$) at all sites passing the site-specific coverage thresholds outlined above were taken to constitute the haplotype. Further methodological details on QPing can be found in Garud & Good (2019). In total, our analyses were performed on 2641 QP haplotypes belonging to the largest clade of each of species considered, as seen in Figure **S9**.

3.5 Inclusion criteria

Once samples had been quasi-phased, we included only species which had both a sufficient number of non-closely related ($d > 5 \times 10^{-4}$) haplotypes and a sufficient number of common variants to allow us to examine LD patterns with high precision. In particular, we included all species which had at least 20 non-closely related samples and at least 10,000 intermediate frequency SNPs (minor allele frequency $f \geq 0.2$). In total, 30 species passed these thresholds.

To perform an iLDS scan, we further required that the reference genome used by MIDAS for a given species was not fragmented into a large number of contigs, each containing only a relatively small number of common, non-synonymous SNVs. In particular, we required that at least one contig have more than 2500 common, non-synonymous SNVs in order for an iLDS scan to be performed. A total of 23 (out of 30 species overall) met this criterion.

3.6 Population structure

Several of the gut microbial species examined here exhibit strong population structure [15, 1, 9], with 'clades' of lineages that recombine far more frequently with one another than with lineages belonging to other clades. In species with strong clade structure, LD is elevated genome-wide and

shows comparatively little decay with distance [1, 9]. Genetic diversity is therefore dominated by fixed differences between clades, masking signatures of ongoing recombination within clades. To control for population structure, we analyzed LD only among lineages belonging to the largest clade (as identified previously in [1]) of each species.

Additionally, we controlled for the potential effects of elevated LD due to the presence of occasional closely related lineages, as measured by the per base-pair divergence d at fourfold degenerate sites (that is, sites where a nucleotide difference does not result in an amino acid change). Pairwise divergence d was determined by first calculating the number of fourfold degenerate sites at which two haplotypes differed, and then dividing this number by the total number of such sites in the core genome. While on average random pairs of lineages belonging to the same clade have d on the order of 10^{-2}/bp, unrelated hosts occasionally harbor very closely related lineages, with pairwise d two orders of magnitude smaller than typically observed across hosts [1, 9]. To avoid elevations in LD arising from these anomalously closely related lineages, we choose only a single representative for any group of lineages $d < 5 \times 10^{-4}$, as in our previous analysis [1].

4 iLDS

4.1 Determining the window size for computing iLDS

To perform an iLDS scan, LD is calculated in sliding windows across the genome, and LD measurements within each window subsequently binned by genomic distance between variants, so that AUC(r^2_N), AUC(r^2_S), and AUC(r^2) within each window may be calculated. To ensure that all windows contained both an adequate and comparable number of synonymous and non-synonymous variants with which to calculate r^2_N and r^2_S curves, windows were defined in terms of SNPs rather than base-pairs. Specifically, each window was defined by the region spanning n consecutive non-synonymous, intermediate frequency SNPs. The value n was chosen such that the average size, in base pairs, of all windows corresponded as closely as possible to the distance over which LD fully decays genome-wide for that species. To determine AUC($r^2_{genome-wide}$) in each window, we first downsampled to 1,000,000 LD measurements total, and then binned these measurements using the bins defined for that window.

To infer the distance over which LD decays, we model recombination as moving tracts of DNA from a donor genome into a recipient genome, where they replace homologous segments in a process known as homologous recombination in bacteria. While bacteria exchange genomic material in a variety of ways, including through the transfer of mobile genetic elements such as plasmids, homologous recombination is thought to be important in breaking up linkage in the core genome, which is the focus of this work [9, 10]. The size of these homologously exchanged tracts vary, but numerous experimental studies have found that, within a species, tract lengths are approximately exponentially distributed [16, 17, 18, 10]. The speed at which loci are unlinked therefore depends crucially on the average size of these recombinant fragments (l_r), which in turn characterizes the total (exponential) distribution of tract lengths. In order to obtain the length scale on which LD decays (l_{DD}), we will first obtain an estimate of l_r, the average tract length.

In our model, two loci recombine when one or the other of the sites, but not both simultaneously, is homologously replaced by an imported fragment. Assuming a uniform per base-pair rate r at which a recombination event may begin at each site along the genome, and an exponential distribution of tract lengths with average length l_r, the effective recombination rate R between loci separated by a distance l is

$$R(l) = rl_r(1 - e^{-l/l_r}) \tag{S5}$$

This functional form suggests that the effective recombination rate increases linearly as $\sim rl$ at short distances (specifically, when $l << l_r$), and ultimately saturates to a single value rl_r for pairs of variants separated by $l >> l_r$. The transition between these two regimes occurs when $l \approx l_r$.

In a neutrally evolving, panmictic population of size N, a classic result [19] states that expected LD obeys the following relationship:

$$E[r^2] = \frac{10 + 2NR}{22 + 26NR + 4(NR)^2} \tag{S6}$$

where R is the effective recombination rate between sites, provided this expectation is taken with respect only to intermediate frequency alleles (e.g. $0.1 < f < 0.9$ [19]). The ultimate cause of the decay of LD is the dependence of the effective recombination rate R on genomic distance—in our model, this relationship is given by Equation S5. We should in principle be able to estimate the parameters N, r, and l_r by fitting Equation S6 to the empirical LD decay curves for intermediate frequency synonymous variants (r_S^2). To obtain an estimate of the effective distance over which LD fully decays—and thereby a window size for iLDS—we are primarily interested in determining l_r.

While Equation S6 describes the behavior of $E[r^2]$ over all distances, its value is, in theory, independent of l_r over short distances ($l << l_r$) as $R(l)$ is itself independent of l_r in this regime. Therefore, as our goal is first to estimate l_r, we disregard short-distance LD and focus only on fitting when $l \approx l_r$ and greater. Using a completely different methodology, a previous study investigating the dynamics of recombination in human gut bacteria (Liu & Good (2023)) estimated l_r for many of the species under consideration here, and obtained values which were $O(10^3) - O(10^4)$. Therefore, we restrict our attention to $l > 10^3$ bp.

In particular, we fit our r_S^2 curves with the following model:

$$E[r^2(l)] = C\frac{10 + 2NR(l)}{22 + 26NR(l) + 4(NR(l))^2} \tag{S7}$$

where C is a normalization constant allowing us to fit the relative change in r_S^2 with distance, rather than absolute values of LD strictly. Fits were obtained using the scipy.optimize.curve_fit function in scipy [20].

To assess the performance of our model visually, we show the empirical r_S^2 and the fit obtained from Equation S7 in Figure **S17**. Overall, we see that the theoretical prediction (Equation S7) provides an excellent fit of LD across several orders of magnitude in the vast majority of species.

Having estimated l_r, we next move to the main goal of inferring l_{DD}, the distance over which LD fully decays genomewide. In our model of homologous recombination, the effective rate of

recombination (and by extension, the level of LD) saturates in the $l >> l_r$ limit of Equation S5—that is, as the distance between variants becomes large relative to the size of a tract that can be horizontally transferred. To infer this distance, we took the value of l_r estimated above as the mean of an exponential distribution of tract lengths for each species. We then took the 99th percentile of this distribution to be our estimate of l_{DD}.

As expected, the decay of LD appears to saturate to a constant value at large distances in all species examined (Figure **S17**). Moreover, by visual inspection, our estimated values of l_{DD} furnish a good approximation for this distance. The estimates of l_r and l_{DD} can be found in Table S3.

Next, to determine the window size n given l_{DD}, we calculated the median number of intermediate frequency non-synonymous SNPs spanning this distance. In general, this window size depends on both l_{DD} and the density of common non-synonymous SNPs, with larger l_{DD} and higher densities leading to greater n.

Finally, once the window size has been assessed, we determined the number of bins b used to bin LD within each window. We set b such that each bin would contain approximately 50 LD measurements—that is, $b = \binom{n}{2}/50$. To avoid creating windows with either very few bins (which obscures the distance dependence of LD within a window) or very many bins (which reduces statistical power), we set a minimum bin number of 3 and a maximum bin number of 10. Window sizes and bin numbers for each species are summarized in Table S3.

4.1.1 Comparison of tract length estimates with previous work

To assess the performance of our method for estimating the mean tract length (l_r) and the 99th percentile of the tract length distribution (l_{DD}), we compared our inferences of these quantities with those of Liu & Good (2023) [9]. The authors of this prior work explored the dynamics of homologous recombination in the same cohort of metagenomic samples examined here, employed QPing to obtain sample-specific haplotypes, and clade-controlled in an identical fashion. While the method developed by Liu & Good (2023) detects recent transfers using pairwise comparisons between closely related strains, we infer statistical properties of the recombination process using the decay of LD among all non-closely related strains. Our approaches are complementary, and below we show a broad agreement between our estimates.

Liu & Good (2023) identified ≈250000 unique instances of recombination—that is, the horizontal exchange of a genomic tract—across 29 species. The tract lengths for each these instances were provided in their supplemental Table S3. For each species analyzed in both datasets (28/29),

we then computed the mean tract length (l_r) and the 99th percentile of the tract length distribution (corresponding to our l_{DD}) from the Liu and Good empirical observations, and compared these quantities with those we obtained using our LD based method.

Overall, we found a correspondence between the two methods (Figure **S19**). Across all shared species, we found a correlation coefficient r^2 of 0.58 for l_r and 0.6 for l_{DD} between our approaches. The species for which our differing methodologies conflict most strongly is *Bacteroides caccae*, which Liu & Good (2023) noted appears to have an anomalously low rate of recombination and large number of closely related lineages. Removing this single species increases the correlation coefficient r^2 for l_r to 0.7 and l_{DD} to 0.65.

4.2 Identification of analysis windows with significant iLDS values

iLDS was applied to the data in analysis windows as defined above. As stated above, iLDS was calculated in sliding windows across the genome, with each window centered around a single intermediate frequency non-synonymous SNVs. iLDS was considered to be significant in a given analysis window if both AUC(r^2_N) significantly exceeded AUC(r^2_S) within the window, and overall linkage AUC(r^2) within the window significantly exceeded the genome-wide expectation for identically sized regions AUC($r^2_{\text{genome-wide}}$). Significance was determined by constructing confidence intervals (as described in the LD Confidence Interval section, above) around r^2_N and r^2 and checking if their lower CIs exceeded r^2_S and $r^2_{\text{genome-wide}}$, respectively, at the $\alpha = 99\%$ level.

4.3 Testing iLDS on simulations

To test the capacity of iLDS to correctly detect selective sweeps, we calculated the statistic on our simulations of sweeps and population contractions. Like sweeps, contractions generate increases in LD, and are known to produce false positive inferences of sweeps in a variety of existing methodologies for detecting selection [21, 22, 23]. Our aim therefore was to establish what percentage of the time iLDS was significant when calculated on sweep simulations (the True Positive Rate: TPR) and what percentage of the time it was significant when calculated on contraction simulations (False Positive Rate: FPR). Furthermore, we sought to also establish how the TPR and FPR depended on the underlying evolutionary parameters: s_D, s_B, ρ, and the magnitude and duration of the contraction.

In real data, iLDS is computed in windows across the genome, and overall linkage within

each window (AUC(r^2)) is compared with the genome-wide average for identically sized windows (AUC($r^2_{genome-wide}$)). Therefore, to calculate the TPR, we first computed r^2_N, r^2_S, and r^2 in the simulated populations undergoing a selective sweep of strength s_B and experiencing purifying selection of strength s_D (Section 2.3). We then compared the elevation in linkage (AUC(r^2)) of each simulated sweep relative to the "burn-in" populations subject to the same strength of purifying selection but lacking a selective sweep. AUC($r^2_{genome-wide}$) was determined by calculating LD in the burn-in populations. Second, to calculate the FPR, we computed r^2_N, r^2_S, and r^2 in simulations where the population had undergone a contraction and was also experiencing purifying selection of strength s_D (Section 2.2). Once again, we compared the elevation in linkage (AUC(r^2)) with the AUC($r^2_{genome-wide}$) determined from the same set of 'burn-in' simulations that were used for selective sweeps.

We chose to calculate AUC($r^2_{genome-wide}$) from the burn-in simulations to mimic genome-wide average LD in a population at mutation-selection balance experiencing the same strength of purifying selection s_D. This allows us to compare the relative rates of significance under sweeps or contractions given the same baseline patterns of genome-wide LD. While selective sweeps lead to local elevations in linkage surrounding the selected site, contractions affect the entire genome. In calculating iLDS, one might therefore plausibly compare LD in each contraction relative to LD in all other contractions of the same magnitude and duration. However, patterns of linkage are known to vary considerably across the genome of populations experiencing a contraction, with some regions exhibiting abnormally high LD and other regions appearing to be at equilibrium [21]. To avoid potentially underestimating the increase in LD for a given contraction, we conservatively chose to compare contraction simulations with the burn-in populations subject to the same strength of purifying selection (but prior to undergoing a contraction) when calculating iLDS, rather than to other simulations of contractions.

Because we simulated relatively short genomic tracts (10Kb), most simulations had only a few intermediate frequency non-synonymous variants following either the sweep or contraction. Consequently, it was not possible to calculate an r^2_N curve for many individual simulations (particularly simulations for which $s_D > 1/N_e$). Moreover, in our empirical scans, iLDS is not calculated on basepair defined windows, but rather on SNP defined windows—in particular, all windows for a given species are pre-determined to have an equal number of intermediate frequency non-synonymous variants. Therefore, to both allow for a sufficient number of LD measurements to calculate r^2_N curves and to replicate the procedure actually used to calculate iLDS empirically, we

merged individual simulations. Specifically, we first calculated LD among synonymous and non-synonymous variants in each simulated population separately, and then concatenated LD measurements belonging to a set number of different simulations of the same type to form a single "merged" population. The LD measured in this merged population represents an average LD across the component simulations. We determined the number of individual simulations to be merged to produce a single merged population by requiring that at least 100 non-synonymous LD measurements be present in each merged population (equivalent to a window size of $\sim$15 SNPs, which is on the low end of window sizes observed in the data.) We then obtained 1000 merged populations by sampling the 1250 simulations of each scenario with replacement 1000 times total.

Overall, we saw that iLDS was significant in a large percentage of positive selection cases when $N_e s_D > 1$ and $s_B > s_D$ (Figure **S7**), indicating that the statistic is able to recover true positives. Indeed, when $s_B = 10^{-1}$ and $s_D = 10^{-2}$ (the strongest selective regime), the TPR was 100%. Demographic contractions, by contrast, had an FPR of zero in nearly all cases. The only regime in which false positives did occur—with a maximum rate of $\approx$1% (Figure **S8**)—was when ρ was very low (10^{-7}) and $s_D < 1/N_e$. As discussed in the Discussion section of the Main Text, these conditions are very unlikely to obtain in the gut microbial species which we analyzed, leading us to conclude that very few, if any, of the selective sweeps detected in the data are a result of demographic contractions. Moreover, far more than 1% of windows were significant in our scans (typically order 10%), so even under this worst case low recombination, weak purifying selection scenario it is unlikely that all or even most windows are false positives.

4.4 Peak calling

After significant windows were identified, we next sought to localize individual gene-specific selective sweeps by identifying "peaks" of elevated values of iLDS. To do so, we identified groups of physically proximate and tightly linked intermediate-frequency non-synonymous SNPs in the center of significant windows—that is, groups of non-synonymous SNPs consistent with having been carried together to intermediate frequency on shared recombinant tracts. Each SNP contained within a peak may be involved in the putative sweep as either a driver or passenger mutation.

We defined tight linkage as pairwise $r^2 \geq 0.5$ between SNPs. Among all pairwise LD comparisons between common, non-synonymous variants within distance l_{DD}, only 1.3% in R. bromii exceeded $r^2 = 0.5$, with similar proportions observed in other species. Thus, our linkage threshold ensures that variants within a peak are among the most tightly linked over this length scale.

To call peaks, we employed a greedy clustering algorithm which grouped tightly linked ($r^2 \geq 0.5$) and physically proximate (distance $\leq l_{DD}$) central SNPs in significant windows. First, we identified all pairwise comparisons between central SNPs in significant windows which met the linkage and proximity criteria described above. Next, we constructed a graph where nodes were individual central SNPs, and edges were drawn between tightly linked, proximate central SNPs. We then extracted the connected component containing the window with the largest iLDS value from this graph. Only connected components with more than one significant window were considered, otherwise solo significant windows were discarded from further peak-calling analysis. By filtering out clusters made up of only a single significant window, we conservatively ensure that all peaks are supported by multiple windows. While not all windows within a peak need be consecutive to one another, and central SNPs need not be tightly linked to all other variants in the peak, each is connected by an edge to at least one other significant window within the peak.

After identifying the peak with the highest iLDS value, we next removed from further peak calling analysis any significant windows within 25Kb of any variant in the cluster. By doing so, we attempt to identify peaks that comprise independent selective events.

We then repeated this procedure—i.e. drawing our proximity/linkage graph, identifying the connected component containing the window with the highest iLDS value as a peak, and removing windows within 25Kb of subsequent peaks—until all significant windows are either placed within a peak or removed from analysis.

To assign putative functions associated with each potential sweep, we then inspected the gene annotations for all significant SNPs contained in a single peak. We report all such annotations in Table S4.

5 Gene enrichment analysis

To assess if particular categories of genes were enriched for signatures of selection, we used gene annotations from the PATRIC database and performed several gene enrichment analyses on different classes of annotations [24, 25]. First, we collated a list of all genes which lay within a peak in any of the 20 species for which we ran a scan (Table S4). Next, we performed separate enrichment analyses on three types of annotations, (1) gene ontology (GO) annotations, (2) predicted gene products, and (3) predicted pathways. We found that a number of gene product classes, including two-component sensor system histidine kinases (8 genes) and SusC/SusD polysaccharide utilization loci (5 SusD, 4 SusC genes under selection) were found to be under selection multiple times (Table S4). Likewise, we found that 13 unique genes lying in starch and sucrose metabolism pathways and 3 unique genes lying in T-cell receptor signaling pathways were under selection. However, when we performed a Fisher exact test comparing the number of these classes of genes detected to be under selection with the number of such genes found among all core genes, and corrected for false discovery using the Benjamini-Hochberg procedure, we found that no GO annotation, gene product, or pathway was significantly overrepresented at the $\alpha = 95\%$ level.

Next, to see if there were more fine-grained patterns were present, we split each gene product into its component words, filtering out all words of three letters or less to remove . We then used the method described above (Fisher exact test, Benjamini-Hochberg correction for false discovery) to check if certain words were overrepresented among genes under selection, relative to their frequency overall in core genes, accounting for FDR. We found only a single word was statistically enriched in genes under selection at the $\alpha = 5\%$ level after FDR correction: maltodextrin (p-value $= 10^{-5}$ pre-correction, adjusted p-value $= 0.048$ post-correction).

6 Analyses with isolates

To augment our analyses using quasi-phased genomes, we downloaded alignments from Unified Human Gastrointestinal Genome collection [6] for *R. bromii* and *C. difficile*. While this dataset contains both isolates and MAGs, we chose to focus exclusively on isolates to minimize potential assembly artifacts. We wrote custom scripts to annotate each SNV falling in a coding region as synonymous or non-synonymous by using coordinates for the reading frame. Metadata for these samples, including accession numbers, can be found in Table S5.

Next, following the pipeline developed for metagenomic samples, we identified top-level clades for each species we analyzed in UHGG by creating a UPGMA dendrogram of all samples using d at 4D sites as a distance metric, and then manually cut the dendrogram if there appeared to be multiple deep clades. As with HMP, we focused our subsequent analyses only on samples belonging to the largest clade identified, and retained only a single representative from each group of closely related samples ($d < 5 \times 10^{-4}$/bp).

For each species in our analyses with QP genomes, we first compiled a list of genes which were a) not inferred to be shared across species boundaries and b) clade-specific core genes (Section 3.3.2) before running downstream analyses on all sites lying within a core gene with sufficient coverage in more than 50% of samples. As the UHGG samples we analyzed were all isolates, it was unnecessary to filter out genes potentially spuriously shared across species boundaries as isolates do not suffer from the issues of read stealing and donating characteristic of metagenomic samples. Instead, we limited our attention (as with the QP genomes) to prevalent sites, defined as sites present in more than 50% of samples.

6.1 Overlap of peaks in the *R. bromii* scans performed in metagenomic QP data and UHGG data

To assess the replicability of our findings, we tested if genes found to be under selection in *R. bromii* using QP genomes were also found to be under selection in the UHGG dataset. To do so, we performed an iLDS scan on the 20 *R. bromii* non-closely related ($d > 5{*}10^{-4}$/bp at 4D sites) isolates found in UHGG. While the metagenomic QP *R. bromii* genomes were largely derived from North America and European metagenomic samples, 17 of 20 UHGG *R. bromii* isolates were Chinese in origin. Despite this, the tract length l_{rr} inferred for the UHGG isolates (2743 bp) closely matched that of the QP genomes (3228 bp).

Next, we blasted all genes found within a peak in metagenomic QP scan for *R. bromii* against all genes found within a peak in the UHGG scan, and considered genes to match if 95$\%$ of the gene aligned with an e-value $< 10^{-10}$.

7 Analyses with *Drosophila melanogaster*

Though the primary focus of this work is detecting selective sweeps in bacteria, hitchhiking of linked deleterious variants with selected sites is a general evolutionary phenomenon that many species experience [26, 27, 28, 29, 30]. If hitchhiking is an important factor elevating r_N^2 above r_S^2, we expect this signal to manifest in a very broad range of adapting populations. Therefore, in addition to our scans in gut bacteria, we further assessed the capacity of iLDS to recapitulate known sweeps in natural populations of *Drosophila melanogaster*, focusing in particular on three loci implicated in pesticide and virus resistance on chromosomes 2R (*Cyp6g1*) and 3R (*Ace*, *CHKov1*) [31, 32, 33, 34].

We downloaded alignments of 100 *D. melanogaster* genomes from the Drosophila Genetics Reference Panel [35] belonging to the Raleigh population, as well as annotations for all sites. While the method for obtaining a window size for iLDS used for bacteria is not appropriate for diploid species (in which recombination is governed by crossing-over, rather than exchange of tracts), we visually estimated that linkage among common synonymous variants fully decayed by $\approx$300Kb on both chromosomes, and used this distances to determine the window sizes on these chromosomes (Figure **S20**). We found that our scans were sharply peaked around the three known instances of selective sweeps at *Cyp6g1*, *Ace*, and *CHKov1* (Figure **S21**). While the sweeps at *Cyp6g1* and *CHKov1* are both driven by transposable element insertions, the sweep at *Ace* is a result of three single nucleotide mutations in that gene [31, 32]. When we applied our peak-calling procedure, in which groups of significant windows centered around variants which are both tightly linked and physically close to one another are considered a peak (Section 4.4), we found that the peak in chromosome 3R near *Ace* (Figure **S21**) was in fact made up of two variants lying in *Ace* and one other nearby variant. However, the peaks lying near *Cyp6g1* in chromosome 2R and *CHKov1* in chromosome 3R did not contain any SNPs lying in these genes, consistent with these sweeps being driven by transposable element insertions which are not directly visible to iLDS, which computes LD and calls peaks using SNPs alone.

While we only tested iLDS's ability to identify simulated single mutational origin selective sweeps (hard sweeps), the sweeps at *Cyp6g1*, *Ace*, and *CHKov1* are all thought to involve multiple, independent mutational origins (soft sweeps) [31, 32]. The ability of iLDS to successfully identify these soft sweeps may indicate that the statistic has power to identify both hard and soft selective sweeps. Future work testing the capacity of iLDS to uncover selective sweeps under a range of adaptive scenarios—including hard versus soft sweeps—is needed.

References

[1] N. R. Garud et al. “Evolutionary dynamics of bacteria in the gut microbiome within and across hosts”. In: *PLoS Biology* 17.1 (2019), e3000102.

[2] J. Lloyd-Price et al. “Strains, functions and dynamics in the expanded Human Microbiome Project”. In: *Nature* 550.7674 (2017), pp. 61–66.

[3] H. Xie et al. “Shotgun metagenomics of 250 adult twins reveals genetic and environmental impacts on the gut microbiome”. In: *Cell Systems* 3.6 (2016), pp. 572–584.

[4] J. Qin et al. “A metagenome-wide association study of gut microbiota in type 2 diabetes”. In: *Nature* 490.7418 (2012), pp. 55–60.

[5] K. Korpela et al. “Selective maternal seeding and environment shape the human gut microbiome”. In: *Genome Research* 28.4 (2018), pp. 561–568.

[6] A. Almeida et al. “A unified catalog of 204,938 reference genomes from the human gut microbiome”. In: *Nature Biotechnology* 39.1 (2021), pp. 105–114.

[7] C. R. Harris et al. “Array programming with NumPy”. In: *Nature* 585.7825 (Sept. 2020), pp. 357–362. DOI: 10.1038/s41586-020-2649-2. URL: https://doi.org/10.1038/s41586-020-2649-2.

[8] B. C. Haller and P. W. Messer. “SLiM 4: multispecies eco-evolutionary modeling”. In: *The American Naturalist* 201.5 (2023), E127–E139.

[9] Z. Liu and B. H. Good. “Dynamics of bacterial recombination in the human gut microbiome”. In: *bioRxiv* (2022), pp. 2022–08.

[10] M. Foerster et al. “Genome-wide transformation reveals extensive exchange across closely related Bacillus species”. In: *bioRxiv* (2023), pp. 2023–07.

[11] J. A. Garcia and K. E. Lohmueller. “Negative linkage disequilibrium between amino acid changing variants reveals interference among deleterious mutations in the human genome”. In: *PLoS Genetics* 17.7 (2021), e1009676.

[12] “A framework for human microbiome research”. In: *Nature* 486.7402 (2012), pp. 215–221.

[13] S. Nayfach et al. “An integrated metagenomics pipeline for strain profiling reveals novel patterns of bacterial transmission and biogeography”. In: *Genome Research* 26.11 (2016), pp. 1612–1625.

[14] B. Langmead and S. L. Salzberg. "Fast gapped-read alignment with Bowtie 2". In: *Nature Methods* 9.4 (2012), pp. 357–359.

[15] P. I. Costea et al. "Subspecies in the global human gut microbiome". In: *Molecular Systems Biology* 13.12 (2017), p. 960.

[16] N. J. Croucher et al. "A high-resolution view of genome-wide pneumococcal transformation". In: *PLoS Pathogens* 8.6 (2012), e1002745.

[17] J. C. Mell et al. "Extensive cotransformation of natural variation into chromosomes of naturally competent Haemophilus influenzae". In: *G3: Genes, Genomes, Genetics* 4.4 (2014), pp. 717–731.

[18] J. J. Power et al. "Adaptive evolution of hybrid bacteria by horizontal gene transfer". In: *Proceedings of the National Academy of Sciences* 118.10 (2021), e2007873118.

[19] G. A. McVean. "A genealogical interpretation of linkage disequilibrium". In: *Genetics* 162.2 (2002), pp. 987–991.

[20] P. Virtanen et al. "SciPy 1.0: fundamental algorithms for scientific computing in Python". In: *Nature Methods* 17.3 (2020), pp. 261–272.

[21] A. Koropoulis, N. Alachiotis, and P. Pavlidis. "Detecting positive selection in populations using genetic data". In: *Statistical population genomics* (2020), pp. 87–123.

[22] J. K. Pritchard and M. Przeworski. "Linkage disequilibrium in humans: models and data". In: *The American Journal of Human Genetics* 69.1 (2001), pp. 1–14.

[23] N. Galtier, F. Depaulis, and N. H. Barton. "Detecting bottlenecks and selective sweeps from DNA sequence polymorphism". In: *Genetics* 155.2 (2000), pp. 981–987.

[24] J. J. Gillespie et al. "PATRIC: the comprehensive bacterial bioinformatics resource with a focus on human pathogenic species". In: *Infection and Immunity* 79.11 (2011), pp. 4286–4298.

[25] M. Ashburner et al. "Gene ontology: tool for the unification of biology". In: *Nature Genetics* 25.1 (2000), pp. 25–29.

[26] S. Chun and J. C. Fay. "Evidence for hitchhiking of deleterious mutations within the human genome". In: *PLoS Genetics* 7.8 (2011), e1002240.

[27] M. Hartfield and S. P. Otto. "Recombination and hitchhiking of deleterious alleles". In: *Evolution* 65.9 (2011), pp. 2421–2434.

[28] M. M. Desai, A. M. Walczak, and D. S. Fisher. "Genetic diversity and the structure of genealogies in rapidly adapting populations". In: *Genetics* 193.2 (2013), pp. 565–585.

[29] C. Steux and Z. A. Szpiech. "The Maintenance of Deleterious Variation in Wild Chinese Rhesus Macaques". In: *bioRxiv* (2023), pp. 2023–10.

[30] Z. J. Assaf, D. A. Petrov, and J. R. Blundell. "Obstruction of adaptation in diploids by recessive, strongly deleterious alleles". In: *Proceedings of the National Academy of Sciences* 112.20 (2015), E2658–E2666.

[31] N. R. Garud et al. "Recent selective sweeps in North American Drosophila melanogaster show signatures of soft sweeps". In: *PLoS Genetics* 11.2 (2015), e1005004.

[32] T. Karasov, P. W. Messer, and D. A. Petrov. "Evidence that adaptation in Drosophila is not limited by mutation at single sites". In: *PLoS Genetics* 6.6 (2010), e1000924.

[33] J. M. Schmidt et al. "Copy number variation and transposable elements feature in recent, ongoing adaptation at the Cyp6g1 locus". In: *PLoS Genetics* 6.6 (2010), e1000998.

[34] M. M. Magwire et al. "Successive increases in the resistance of Drosophila to viral infection through a transposon insertion followed by a duplication". In: *PLoS Genetics* 7.10 (2011), e1002337.

[35] T. F. Mackay et al. "The Drosophila melanogaster genetic reference panel". In: *Nature* 482.7384 (2012), pp. 173–178.
